# Three PilZ Domain Proteins, PlpA, PixA, and PixB, Have Distinct Functions in Regulation of Motility and Development in Myxococcus xanthus

**DOI:** 10.1128/JB.00126-21

**Published:** 2021-06-08

**Authors:** Sofya Kuzmich, Dorota Skotnicka, Dobromir Szadkowski, Philipp Klos, María Pérez‐Burgos, Eugenia Schander, Dominik Schumacher, Lotte Søgaard-Andersen

**Affiliations:** a Department of Ecophysiology, Max Planck Institute for Terrestrial Microbiology, Marburg, Germany; Geisel School of Medicine at Dartmouth

**Keywords:** c-di-GMP, PilZ domain, *Myxococcus*, type IV pili, gliding motility, chemosensory system, Frz, MglA, fruiting body formation, sporulation, MglA GTPase, exopolysaccharide, fruiting body

## Abstract

In bacteria, the nucleotide-based second messenger bis-(3′-5′)-cyclic dimeric GMP (c-di-GMP) binds to effectors to generate outputs in response to changes in the environment. In Myxococcus xanthus, c-di-GMP regulates type IV pilus-dependent motility and the starvation-induced developmental program that results in formation of spore-filled fruiting bodies; however, little is known about the effectors that bind c-di-GMP. Here, we systematically inactivated all 24 genes encoding PilZ domain-containing proteins, which are among the most common c-di-GMP effectors. We confirm that the stand-alone PilZ domain protein PlpA is important for regulation of motility independently of the Frz chemosensory system and that Pkn1, which is composed of a Ser/Thr kinase domain and a PilZ domain, is specifically important for development. Moreover, we identify two PilZ domain proteins that have distinct functions in regulating motility and development. PixB, which is composed of two PilZ domains and an acetyltransferase domain, binds c-di-GMP *in vitro* and regulates type IV pilus-dependent and gliding motility in a Frz-dependent manner as well as development. The acetyltransferase domain is required and sufficient for function during growth, while all three domains and c-di-GMP binding are essential for PixB function during development. PixA is a response regulator composed of a PilZ domain and a receiver domain, binds c-di-GMP *in vitro*, and regulates motility independently of the Frz system, likely by setting up the polarity of the two motility systems. Our results support a model whereby PlpA, PixA, and PixB act in independent pathways and have distinct functions in regulation of motility.

**IMPORTANCE** c-di-GMP signaling controls bacterial motility in many bacterial species by binding to downstream effector proteins. Here, we identify two PilZ domain-containing proteins in Myxococcus xanthus that bind c-di-GMP. We show that PixB, which contains two PilZ domains and an acetyltransferase domain, acts in a manner that depends on the Frz chemosensory system to regulate motility via the acetyltransferase domain, while the intact protein and c-di-GMP binding are essential for PixB to support development. In contrast, PixA acts in a Frz-independent manner to regulate motility. Taking our results together with previous observations, we conclude that PilZ domain proteins and c-di-GMP act in multiple independent pathways to regulate motility and development in M. xanthus.

## INTRODUCTION

Bacteria have evolved different strategies that allow them to sense and subsequently adapt and differentiate in response to changing environmental conditions. One strategy centers on signaling by a variety of nucleotide-based second messengers (1). The highly versatile second messenger bis-(3′-5′)-cyclic dimeric GMP (c-di-GMP) is widespread in bacteria and is involved in many aspects of bacterial physiology, including regulation of motility, adhesion, synthesis of secreted polysaccharides, biofilm formation, cell cycle progression, development, and virulence (2, 3).

Signaling by c-di-GMP depends on its regulated synthesis by diguanylate cyclases (DGCs), which contain an enzymatically active GGDEF domain, and degradation by phosphodiesterases (PDEs), which contain either a catalytic EAL or HD-GYP domain (2, 3). Output responses are generated by binding of c-di-GMP to and allosteric regulation of effectors, which direct downstream responses at the transcriptional, translational, or posttranslational level (2, 3). Several different protein effectors with little sequence homology have been identified and include enzymatically inactive GGDEF and EAL domain proteins (4–8), PilZ domain proteins (9–15), MshEN domain proteins (16, 17), members of different transcription factor families as well as a nucleoid-associated DNA binding protein (18–26), and ATPases of flagella and type III and type VI secretion systems (27). The number of DGCs and PDEs encoded by many bacterial genomes typically exceeds that of known c-di-GMP effectors (2, 28), thus hampering a detailed understanding of how effects of changing c-di-GMP levels are implemented.

Myxococcus xanthus, a Gram-negative deltaproteobacterium, is a model organism for studying how social behaviors in bacteria can be modulated by environmental cues (29, 30). In the presence of nutrients, M. xanthus cells grow, divide, and actively move by means of two motility systems to generate colonies in which cells spread outward in a highly coordinated fashion and prey on other microorganisms if present. In response to nutrient depletion, a developmental program is initiated that results in the formation of multicellular, spore-filled fruiting bodies. Both social behaviors depend on regulated motility (31, 32) as well as signaling by c-di-GMP (33).

The rod-shaped M. xanthus cells move on surfaces in the direction of their long axis using two polarized motility systems and with clearly defined leading and lagging cell poles (31, 32). Gliding motility generally allows the movement of single cells (34) and depends on the Agl/Glt complexes, which assemble at the leading cell pole, attach to the substratum, and disassemble as they reach the lagging cell pole (35, 36). In contrast, type IV pilus (T4P)-dependent motility mostly occurs in groups of cells (34, 37). T4P localize to the leading cell pole and undergo cycles of extension, surface adhesion, and retraction to pull cells across surfaces (38–40). Moreover, T4P-dependent motility in M. xanthus depends on a secreted exopolysaccharide (EPS) (41–45). Occasionally, M. xanthus cells reverse their direction of movement, and these reversals are induced by the Frz chemosensory system (46).

The characteristic polarized assembly of the two motility machineries at the leading pole is regulated by a set of four proteins that make up the so-called cell polarity module. The key protein in this module is the small GTPase MglA, which in its GTP-bound form localizes at the leading cell pole to stimulate assembly of the Agl/Glt complexes as well as formation of T4P (35, 47–50). The activity and localization of MglA are regulated by its cognate guanine nucleotide exchange factor (GEF), composed of the RomR and RomX proteins (51), and its cognate GTPase activating protein (GAP), MglB (47, 50). These three proteins localize in bipolar, asymmetric patterns to the cell poles, with the GAP activity dominating at the lagging cell pole and the GEF activity dominating at the leading cell pole (47, 50–52). Importantly, the MglB GAP activity at the lagging pole blocks MglA-GTP accumulation at this pole and therefore ensures that MglA-GTP stimulates T4P formation only at the leading pole (49). Similarly, because MglA-GTP is incorporated into the Agl/Glt complexes, these complexes disassemble as they reach the lagging pole (35). Consequently, cells lacking MglB undergo frequent Frz-independent reversals because they have T4P at both poles and do not disassemble the Agl/Glt complexes at the lagging pole (35, 49). During Frz-dependent reversals, Frz signaling induces the relocalization of MglA, MglB, RomR, and RomX between the two poles (47, 50–52), thus laying the foundation for T4P formation and Agl/Glt complex assembly at the new leading cell pole after a reversal.

c-di-GMP accumulates during growth of M. xanthus and at a 10-fold-higher level during development (53, 54). During growth, c-di-GMP regulates T4P-dependent motility by regulating transcription of the *pilA* gene, which encodes the major pilin subunit of T4P, and EPS synthesis (7, 53). During development, the increased c-di-GMP level induces an increase in EPS synthesis that is essential for fruiting body formation and sporulation (54). Among the 17 GGDEF domain-containing proteins encoded by the M. xanthus genome, only DmxA and DmxB have been experimentally shown to have DGC activity (53, 54). Lack of DmxA causes defects only during growth and is important for T4P-dependent motility (53). The c-di-GMP receptors involved in regulating *pilA* transcription and EPS synthesis during growth are not known. Lack of DmxB causes defects only during development, and DmxB is responsible for the increase in the c-di-GMP level during development (54). c-di-GMP binds to the transcriptional regulator EpsI/Nla24 (54), which is an enhancer binding protein important for expression of genes encoding proteins for EPS synthesis (55). It was suggested that c-di-GMP may bind to EpsI/Nla24 during development to stimulate EPS synthesis (54). However, generally, it is largely unknown how effects of changing c-di-GMP levels are implemented to affect motility and development.

**FIG 1:**
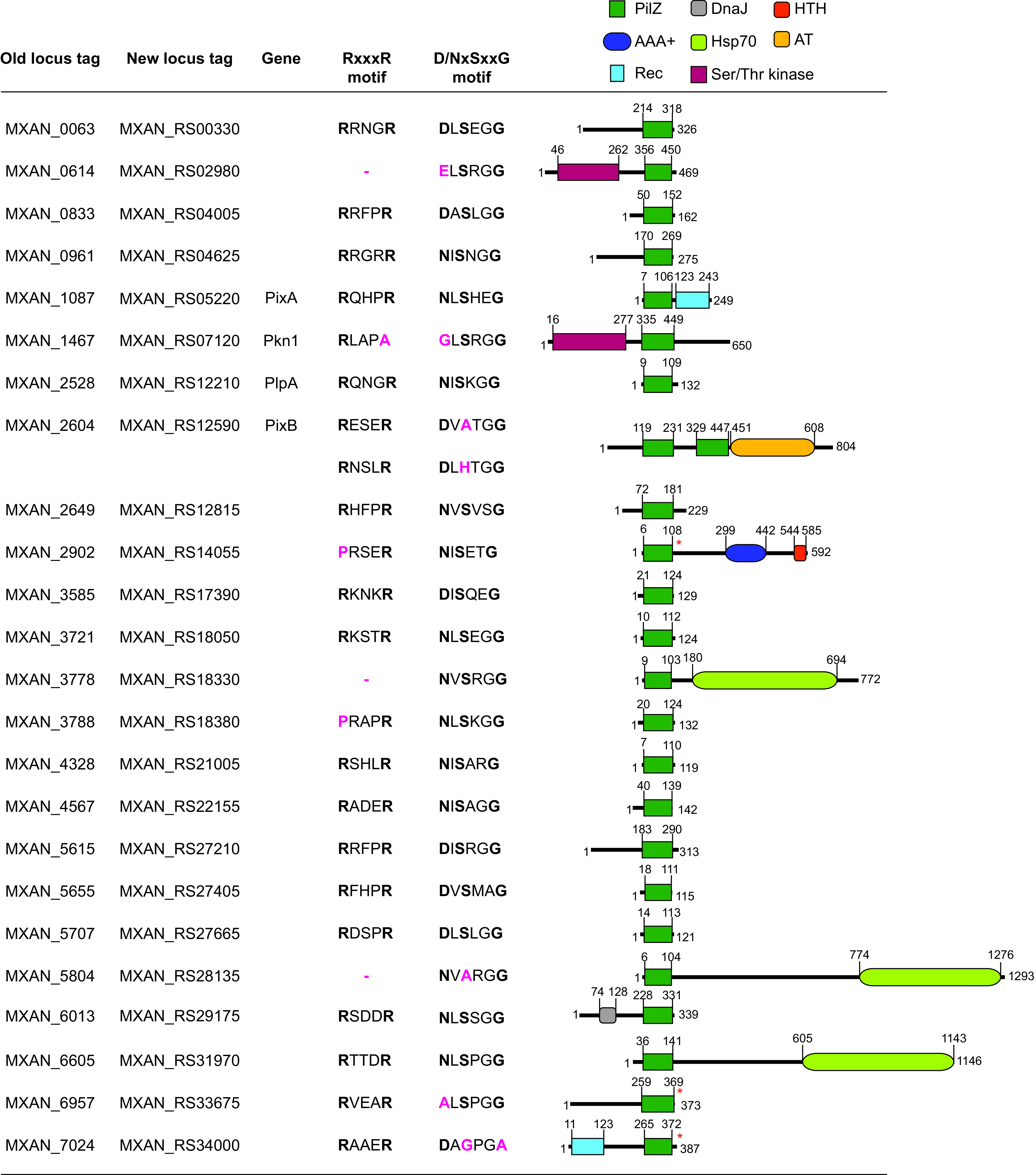
Analysis of M. xanthus proteins containing a PilZ domain. Domain organization of M. xanthus proteins containing a PilZ domain. Locus tags according to the original MXAN annotation of the M. xanthus genome and the most recent annotation are included, together with names of proteins described in the literature (see the text). Protein domains were identified with Pfam, except the PilZ domains marked with a red asterisk, which were identified with Motif Scan (see Materials and Methods). Domains are drawn to scale. The two conserved sequence motifs involved in c-di-GMP binding (2) are indicated at the top, and the corresponding amino acid residues are indicated in black for conserved and pink for nonconserved residues.

Here, we aimed to further understand the molecular basis of how the effects of changing levels of c-di-GMP are implemented in M. xanthus. Because PilZ domain proteins in several species have been shown to be involved in regulation of flagellum-based motility (14, 56–60), T4P-dependent motility (61, 62), and synthesis of secreted polysaccharides (12, 63, 64), we addressed the function of PilZ domain proteins in M. xanthus. Previously, bioinformatics analyses identified 24 PilZ domain proteins in M. xanthus (9), among which only three have been analyzed experimentally. Pkn1 is a Ser/Thr kinase that is important for development (65) (Fig. 1). Based on sequence analysis, the PilZ domain of Pkn1 is predicted not to bind c-di-GMP; however, c-di-GMP binding by Pkn1 has not been studied. PlpA consists of a single PilZ domain (Fig. 1) and is important for regulation of the reversal frequency independently of the Frz system but not for development (66). Despite possessing the conserved residues in the PilZ domain for c-di-GMP binding (Fig. 1), PlpA was reported not to bind c-di-GMP *in vitro*, and substitutions of amino acids predicted to be important for c-di-GMP binding did not cause reversal defects *in vivo* (66). MXAN_2902 is an enhancer-binding protein (Fig. 1) which is important for fruiting body morphology under a subset of starvation conditions (67) and contains a PilZ domain that is predicted not to bind c-di-GMP; however, c-di-GMP binding has not been studied experimentally.

Here, we report the identification of two PilZ domain proteins, renamed PixA (MXAN_1087/MXAN_RS05220 [old/new annotation]) and PixB (MXAN_2604/MXAN_RS12590), for PilZ domain protein in M. xanthus A and B, that both bind c-di-GMP *in vitro*. PixA is a response regulator with a single PilZ domain and involved in regulation of the cellular reversal frequency independently of the Frz system. PixB is composed of two PilZ domains and an acetyltransferase domain. PixB regulates the reversal frequency in a Frz-dependent manner and is also important for development. The acetyltransferase activity is essential for activity and is likely stimulated by c-di-GMP binding to the PilZ domains during development.

## RESULTS

### Phenotypic characterization of mutants lacking individual PilZ domain proteins.

The ∼110-amino-acid PilZ domain is widely distributed in bacteria and can be found either as a stand-alone domain in PilZ single-domain proteins or in combination with other domains (9). Genome analyses previously revealed that the M. xanthus genome encodes 24 proteins with a PilZ domain (9) (Fig. 1; also, see Fig. S1 in the supplemental material). Fourteen are composed only of a PilZ domain, while the remainder contain additional domains. Some of the latter are typically involved in signal transduction, while four proteins contain a DnaK or DnaJ domain, suggesting that they might be involved in protein folding and/or quality control. In fully sequenced *Myxococcales* genomes, the 24 PilZ domain proteins are largely conserved in closely related fruiting *Cystobacterineae* but not in the nonfruiting *Cystobacterineae* and the more distantly related fruiting *Nannocystineae* and *Sorangineae* (Fig. S2A).

**FIG 2:**
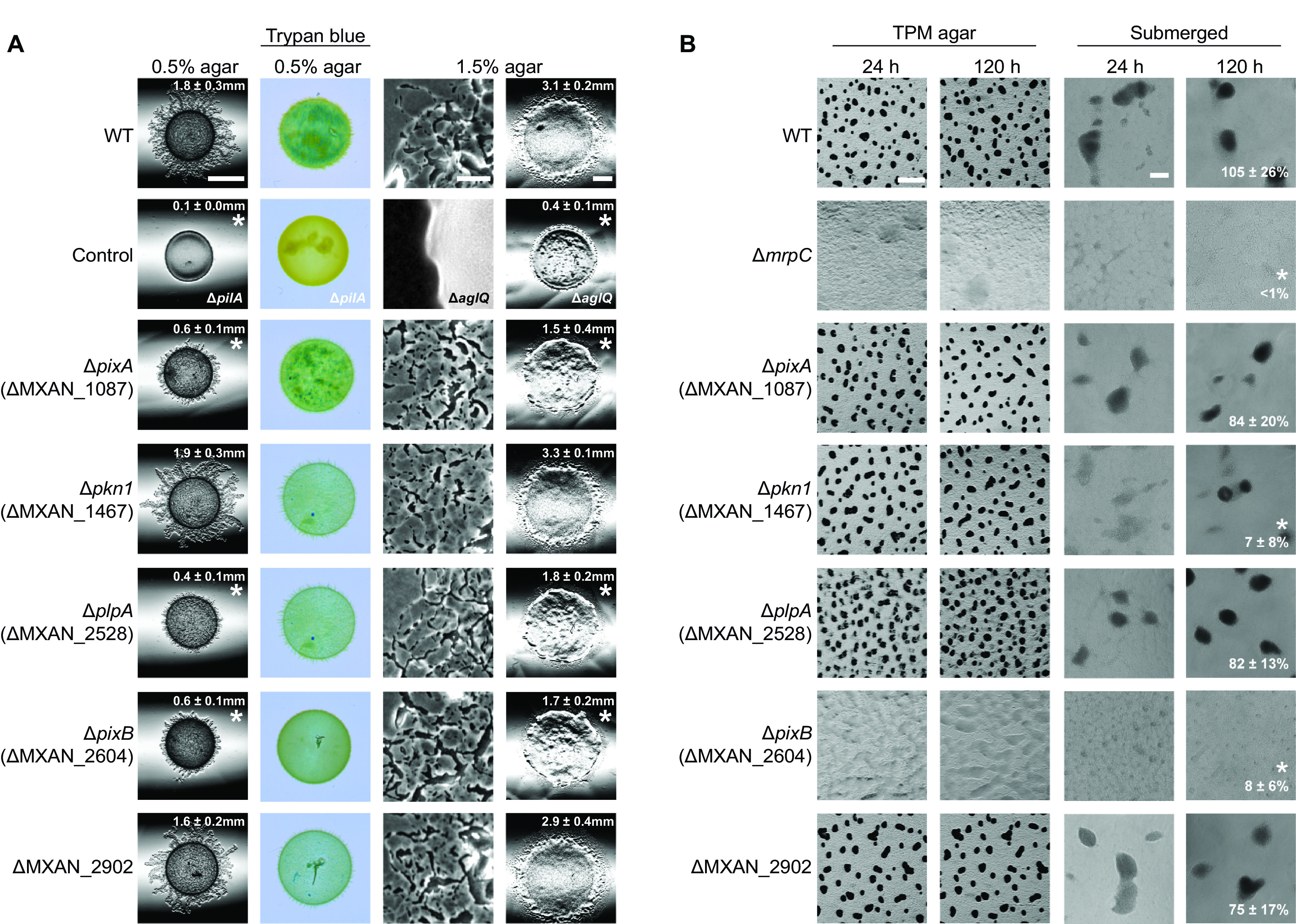
Motility and development assays for mutants lacking PilZ domain proteins. (A) Assays for T4P-dependent and gliding motility. T4P-dependent motility and gliding motility were analyzed on 0.5% agar and 1.5% agar supplemented with 0.5% CTT, respectively. Motility was quantified by the increase in colony radius, and numbers indicate the mean increase in colony radius ± standard deviation (SD) from three biological replicates after 24 h. *, *P < *0.05 in a Student's *t* test. Bars, 3 mm (0.5% agar), 100 μm (1.5% agar, left), and 3 mm (1.5% agar, right). EPS accumulation was determined on 0.5% agar supplemented with 0.5% CTT and 10 μg/ml trypan blue. (B) Fruiting body formation and sporulation on TPM agar and in MC7 submerged culture. Numbers indicate formation of heat- and sonication-resistant spores at 120 h of starvation in submerged culture, given as percentage of WT (100%) ± SD from three biological replicates. *, *P < *0.05 in Student's *t* test. Bars, 500 μm (TPM agar) and 100 μm (submerged).

We systematically generated in-frame deletion mutations in all 24 genes encoding PilZ domain proteins and tested the strains for motility, EPS accumulation (Fig. 2; Fig. S3), and development (Fig. 2; Fig. S4). On 0.5% agar, which is favorable for T4P-dependent motility (37), the wild-type (WT) strain DK1622 formed the long flares characteristic of T4P-dependent motility, while the Δ*pilA* mutant, which lacks the major pilin of T4P, did not (Fig. 2; Fig. S3). Among the 24 mutants generated, the Δ*plpA*, Δ*pixA*, and Δ*pixB* mutants had defects in T4P-dependent motility, with the formation of shorter flares and significantly reduced colony expansion. Because altered EPS accumulation causes reduced T4P-dependent motility, we determined EPS synthesis using a trypan blue-based colorimetric assay. We observed that all 24 mutants synthesized EPS at levels similar to that of the WT, while the level was decreased in the Δ*pilA* mutant, which served as a negative control (68) (Fig. 2). On 1.5% agar, which is favorable to gliding motility (37), the WT displayed the characteristic single-cell movement at the edge of the colony, whereas the Δ*aglQ* mutant, which lacks the motor of the gliding motility complex (69, 70), did not (Fig. 2; Fig. S3). Among the 24 mutants generated, the Δ*plpA*, Δ*pixA*, and Δ*pixB* mutants had defects in gliding, with fewer single cells at the colony edge and significantly reduced colony expansion. These observations are in agreement with previous analyses of a *plpA* mutant (66).

We tested the ability of the 24 generated mutants to undergo development with fruiting body formation and sporulation by starving cells under two different conditions, i.e., on TPM agar and on a polystyrene surface under submerged conditions. Under both conditions, WT cells formed fruiting bodies at 24 h and spores at 120 h, as measured for cells starved under submerged conditions, while the Δ*mrpC* mutant, which lacks a transcription factor important for development and served as a negative control (71), did not (Fig. 2; Fig. S4). Among the 24 mutants generated, only the Δ*pkn1* and Δ*pixB* mutants had developmental defects. In agreement with previous observations (65), the Δ*pkn1* mutant formed fruiting bodies but was reduced in sporulation; the Δ*pixB* mutant did not form fruiting bodies under the two conditions tested and was also reduced in sporulation. We did not observe developmental defects in the ΔMXAN_2902 mutant under the two conditions tested (Fig. 2).

We conclude that the Δ*plpA*, Δ*pixA*, and Δ*pix*B mutations cause defects in both motility systems and the Δ*pkn1* and Δ*pixB* mutations cause defects in development. From this point on, we focused on PixA and PixB.

### PixA and PixB have distinct functions in regulation of motility.

PixA is a response regulator of two-component systems with an N-terminal PilZ domain that possesses all necessary residues for c-di-GMP binding (Fig. 1; Fig. S1) and a C-terminal receiver domain in which the conserved residues important for phosphorylation, including the potentially phosphorylatable Asp180 residue, are conserved (Fig. S5) (72). PixB consists of two PilZ domains that both contain the conserved RXXXR motif for c-di-GMP binding but lack the conserved Ser residue in the second motif (Fig. 1; Fig. S1) and an acetyltransferase (AT) domain of the Gcn5-related *N*-acetyltransferase (GNAT) family. Both proteins as well as the genetic neighborhood of the respective genes are conserved in closely related fruiting *Cystobacterineae* (Fig. S2A to C).

**FIG 3:**
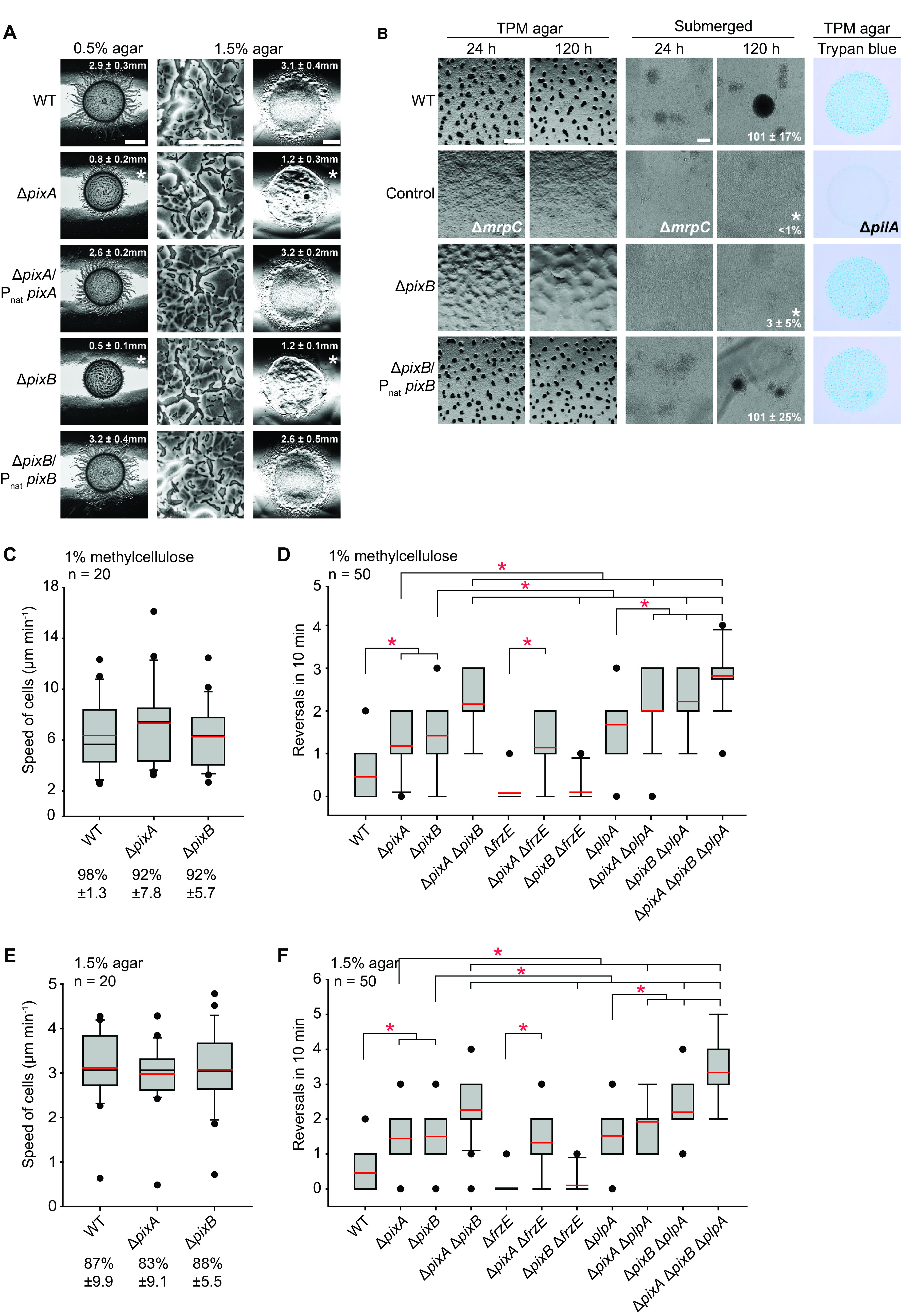
PixA is important for motility and PixB for motility and development. (A) Complementation of Δ*pixA* and Δ*pixB* motility defects. Assays were done as for Fig. 2A. Bars, 2 mm (0.5% agar), 100 μm (1.5% agar, left), and 3 mm (1.5% agar, right). (B) Complementation of Δ*pixB* developmental defects. Fruiting body formation and sporulation were analyzed as for Fig. 2B. EPS accumulation was determined on TPM agar with addition of trypan blue. Numbers indicate formation of heat- and sonication-resistant spores at 120 h of starvation in submerged culture, given as percentage of the WT value (100%) ± SD from three biological replicates. *, *P < *0.05 in Student's *t* test. Bars, 500 μm (TPM agar) and 100 μm (submerged). (C and E) Speed of Δ*pixA* and Δ*pixB* cells in 1% methylcellulose (C) and on 1.5% agar supplemented with 0.5% CTT (E). Box plots show the speed of isolated cells per minute, boxes enclose the 25th and 75th percentiles, whiskers represent the 10th and 90th percentiles, and dots show outliers; red and black lines indicate means and medians, respectively. *, *P < *0.05 in the Mann-Whitney rank sum test; *n* = 20 cells. Numbers below the *x* axis indicate the fraction of cells that displayed single-cell movement (mean ± SD). (D and F) Reversal frequency of Δ*pixA*, Δ*pixB*, and Δ*plpA* mutants on 1% methylcellulose (D) and 1.5% agar (F). Box plots show the reversals of cells per 10 min. Box plots are as described for panels C and E. *, *P < *0.05 in the Mann-Whitney rank sum test; *n* = 50 cells.

The distance between *pixA* and the downstream gene supports the idea that *pixA* is not part of an operon, while a similar analysis of the *pixB* locus supports the idea that *pixB* could be in an operon with the two downstream genes (Fig. S2B and C). To determine whether *pixA* and *pixB* are indeed important for motility and development in the case of *pixB*, we ectopically expressed the full-length genes under the control of their native promoter (P_nat_) from the *attB* site in the Δ*pixA* and Δ*pixB* mutants (Fig. S2B and C). Motility assays on 0.5% and 1.5% agar plates revealed that the motility defects of both mutants were fully corrected in the two complementation strains (Fig. 3A); similarly, the developmental defects of the Δ*pixB* mutant were complemented by ectopic expression of *pixB* (Fig. 3B). Because the increased c-di-GMP level during development is important for EPS synthesis (54), we determined EPS accumulation in developing cells. We observed that the Δ*pixB* mutant accumulated EPS similarly to the WT, as estimated by the trypan blue binding assay (Fig. 3B).

Motility defects scored in a population-based assay can be caused by bona fide motility defects or an altered reversal frequency. To discriminate between these defects, we scored the speed and reversal frequency of single cells moving preferentially by T4P-dependent motility in 1% methylcellulose and by gliding on 1.5% agar in single cell motility assays. Δ*pixA* and Δ*pixB* cells moved with the same speed as WT cells under both conditions (Fig. 3C and E) but reversed significantly more frequently than the WT under both conditions (Fig. 3D and F).

To test whether PixA and PixB regulate cellular reversals in a Frz-dependent manner, we deleted *frzE*, which encodes the FrzE kinase (73), in the Δ*pixA* and Δ*pixB* mutants. The Δ*frzE* mutant as expected had a hyporeversing phenotype under both conditions tested; importantly, the Δ*frzE* Δ*pixA* mutant hyperreversed and the Δ*frzE* Δ*pixB* mutant, similarly to the Δ*frzE* mutant, hyporeversed (Fig. 3D and F). Thus, PixA acts independently of the Frz system to inhibit reversals, while PixB inhibits reversals in a Frz-dependent manner.

Lack of PlpA also causes Frz-independent hyperreversals (66). We used epistasis experiments to address whether PixA, PixB, and PlpA act in the same pathway. We confirmed that the Δ*plpA* mutant hyperreverses; more importantly, all three double mutants had an additive phenotype and reversed more frequently than the three single mutants (Fig. 3D and F). Finally, the triple mutant reversed even more frequently than the three double mutants (Fig. 3D and F).

Altogether, these observations demonstrate that PixA and PixB, similarly to PlpA, are important not for motility *per se* but for cells to move with the correct reversal frequency. The epistasis tests support the idea that these three proteins act in independent pathways to establish the correct reversal frequency.

### PixA is important for unipolar localization of MglA.

**FIG 4:**
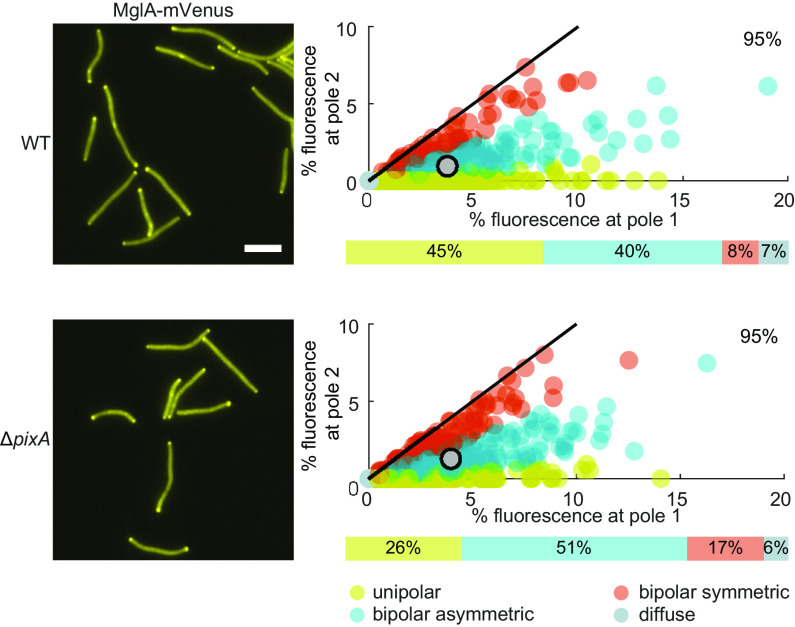
PixA is important for unipolar MglA-mVenus localization. In the scatterplots, the percentage of total fluorescence at pole 2 is plotted against the percentage of total fluorescence at pole 1 for all cells with a polar cluster(s). Pole 1 is by definition the pole with the highest fluorescence. For each cell with polar clusters, an asymmetry index (ω) was calculated as described in Materials and Methods to distinguish between unipolar, asymmetric bipolar, and symmetric bipolar localization; cells with no polar signal were categorized as cells with diffuse localization. The bars below the scatterplots indicate the fractions of cells with a particular localization pattern. Individual cells in the scatterplots are color coded according to their localization pattern. Black lines are symmetry lines, gray dots show means, and numbers in the upper right corners are the mean percentages of total fluorescence in the cytoplasm. *n* = 400 cells from two biological replicates with 200 cells each. Bar, 5 μm.

Frz-independent reversals can be caused by interfering with the correct localization of the proteins of the polarity module. In particular, mutations that cause a more bipolar localization of MglA cause Frz-independent hyperreversals (47, 50, 74). Because a lack of PixA causes hyperreversals independently of the Frz system (Fig. 3D and F), we hypothesized that PixA would be important for correct MglA localization. To test this hypothesis, we expressed an MglA-mVenus fusion protein from the native site in the WT and the Δ*pixA* mutant. Epifluorescence microscopy demonstrated that MglA-mVenus in the absence of PixA localizes in a more symmetric bipolar pattern than in the WT, while the total polar signal remained unchanged (Fig. 4). In agreement with this observation, the distribution of ω values, which provide a measure of MglA localization asymmetry (see Materials and Methods), in the WT and the Δ*pixA* mutant is significantly different (*P < *0.005 in a two-sided *t* test).

A lack of PlpA also causes a shift in MglA-GTP toward symmetric bipolar localization (66). Altogether, these observations together with the epistasis analyzes demonstrate that PixA and PlpA act in independent pathways to establish unipolar localization of MglA.

### PixA and PixB bind c-di-GMP *in vitro*.

**FIG 5:**
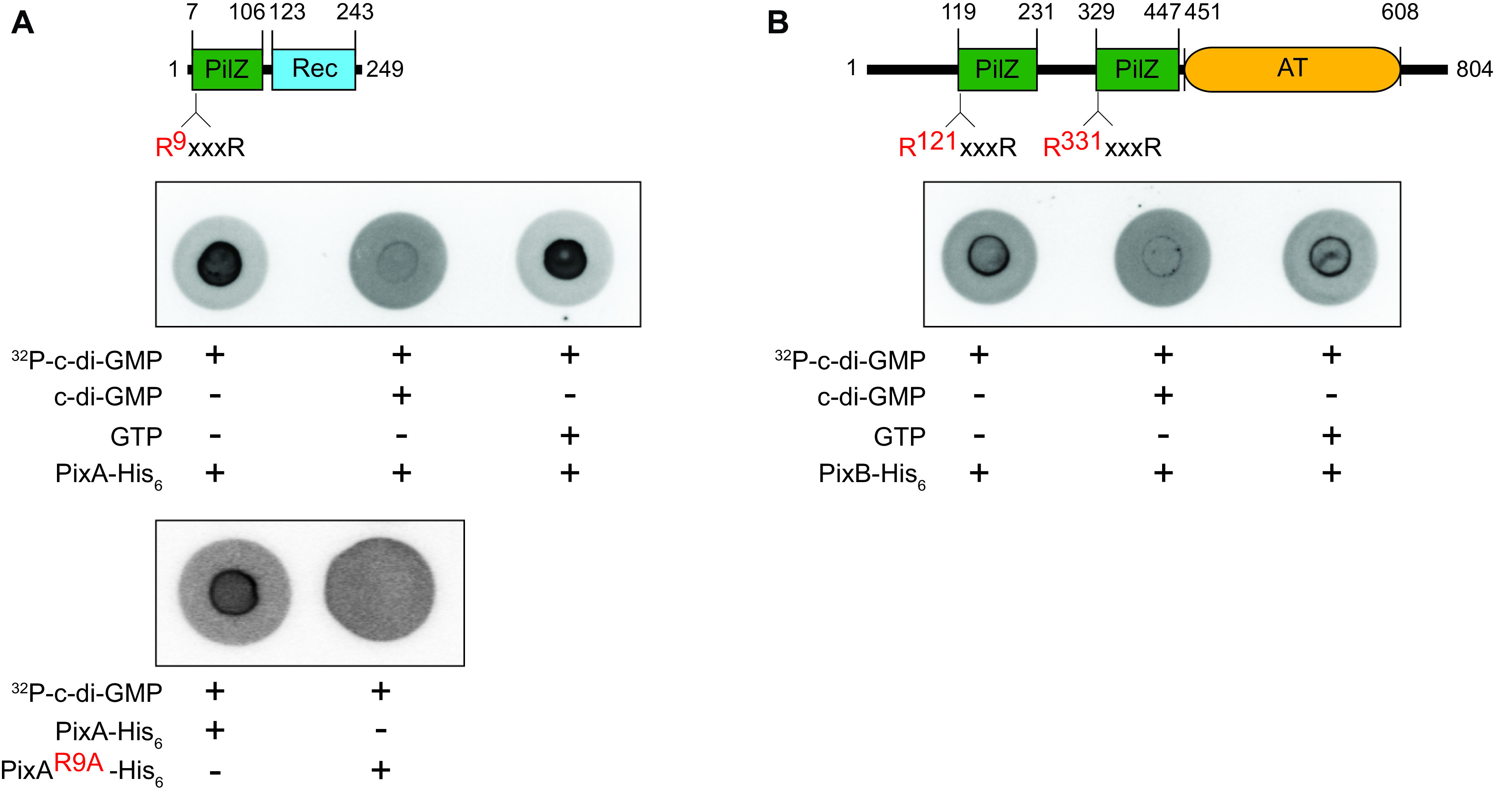
*In vitro* assay for c-di-GMP binding by PixA and PixB. DRaCALA assay to detect specific c-di-GMP binding by purified proteins. Full-length PixA-His_6_ (A, top), PixA^R9A^-His_6_ (A, bottom), and PixB-His_6_ (B) were incubated at a final concentration of 20 μM with ^32^P-labeled c-di-GMP. Unlabeled c-di-GMP and GTP were added to final concentrations of 400 μM, as indicated.

The PilZ domain in PixA contains the two motifs important for c-di-GMP binding while both PilZ domains in PixB have a substitution in the most C-terminal of the two motifs (Fig. 1; Fig. S1). To determine whether PixA and PixB can bind c-di-GMP, we performed a differential radial capillary action of ligand assay (DRaCALA) using purified full-length PixA-His_6_ and PixB-His_6_ (Fig. 5A and B; Fig. S6). PixA-His_6_ and PixB-His_6_ specifically bound ^32^P-labeled c-di-GMP (Fig. 5A and B). As expected, the PixA^R9A^-His_6_ variant, which contains the Arg9-to-Ala substitution in the N-terminal part of the bipartite c-di-GMP binding motif (Fig. 5A; Fig. S6), did not detectably bind [^32^P]c-di-GMP (Fig. 5A). To determine which of the PilZ domains in PixB are involved in c-di-GMP binding, we overexpressed the PixB^R121A^-His_6_, PixB^R331A^-His_6_, and PixB^R121A/R331A^-His_6_ variants, which contain a substitution in the N-terminal part of the c-di-GMP binding motif (Fig. 5B). However, we were not able to purify any of these variants, because they formed inclusion bodies under all conditions tested.

We conclude that PixA binds c-di-GMP *in vitro* and that the R^9^XXXR motif is important for this binding; PixB also binds c-di-GMP *in vitro*, but it is unclear which domain(s) is involved.

### Role of c-di-GMP binding by PixA and PixB *in vivo*.

**FIG 6:**
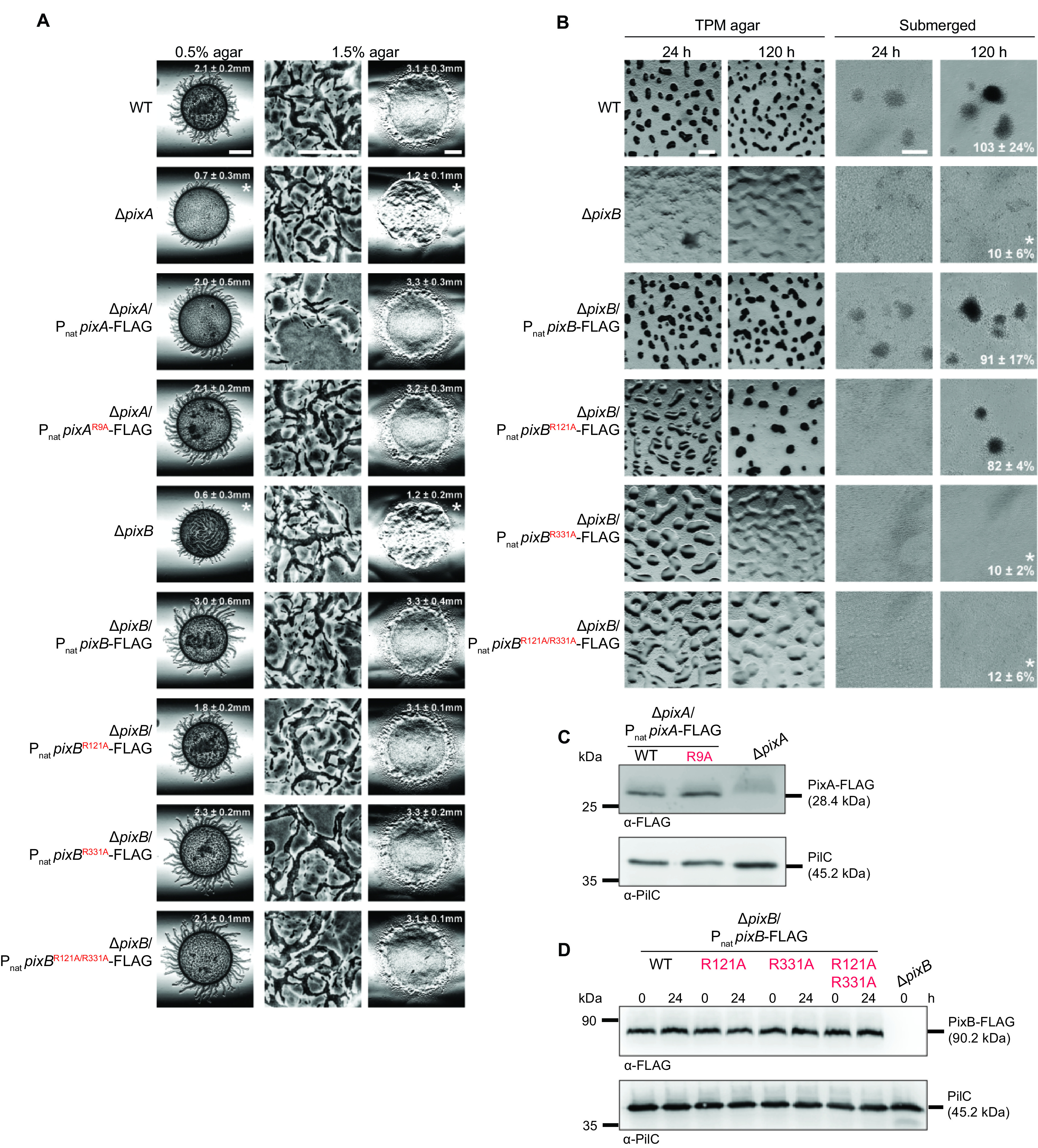
Role of c-di-GMP binding by the PixA and PixB PilZ domains in regulation of motility and development. (A) Motility assays of strains expressing full-length WT or protein variants with a mutated RXXXR motif in the PilZ domains. Motility was analyzed as described for Fig. 2A. Bars, 3 mm (0.5% agar), 100 μm (1.5% agar, left), and 3 mm (1.5% agar, right). (B) Development assays of the Δ*pixB* mutant expressing full-length WT or protein variants with a mutated RXXXR motif in PilZ domains. Assays were done as described for Fig. 2B. Bars, 500 μm (TPM agar) and 100 μm (submerged). (C and D) Immunoblot detection of PixA-FLAG (C) and PixB-FLAG (D). For PixA-FLAG samples, total cell extracts were prepared from exponentially grown cells; for PixB-FLAG samples, cell extracts were prepared from exponentially grown cells and at 24 h of starvation in submerged culture. Ten micrograms of protein was loaded per lane, and samples were separated by SDS-PAGE. Upper blots were probed with anti-FLAG antibodies and lower blots with anti-PilC antibodies. The PilC blots served as loading controls. Molecular mass markers are indicated on the left.

To test whether c-di-GMP binding is important for PixA and PixB function *in vivo*, we ectopically expressed FLAG-tagged full-length WT proteins and mutant variants from the Mx8 *attB* site in the relevant in-frame deletion mutants (Fig. 6A and B). PixA^WT^-FLAG and PixA^R9A^-FLAG accumulated at similar levels during growth (Fig. 6C) and restored motility, including the reversal frequency in the Δ*pixA* mutant (Fig. 6A; Fig. S7A and B).

PixB^WT^-FLAG and the variants PixB^R121A^-FLAG, PixB^R331A^-FLAG, and PixB^R121A/R331A^-FLAG accumulated at similar levels during growth and development and all four proteins restored the motility defects of the Δ*pixB* mutant (Fig. 6A and D; Fig. S7AB). While fruiting body formation and sporulation were fully restored by PixB^WT^-FLAG (Fig. 6B), the strain complemented with PixB^R121A^-FLAG displayed delayed fruiting body formation both on solid agar and under submerged conditions but restored sporulation at 120 h; however, PixB^R331A^-FLAG and PixB^R121A/R331A^-FLAG restored neither fruiting body formation nor sporulation in the Δ*pixB* mutant (Fig. 6B).

These observations support the idea that c-di-GMP binding by PixA and PixB under the conditions tested is not important for their function in motility. In contrast, c-di-GMP binding by the two PilZ domains in PixB appears to be important for fruiting body formation and sporulation, with the C-terminal domain being the most important.

### Structure-function analysis of PixA and PixB.

**FIG 7:**
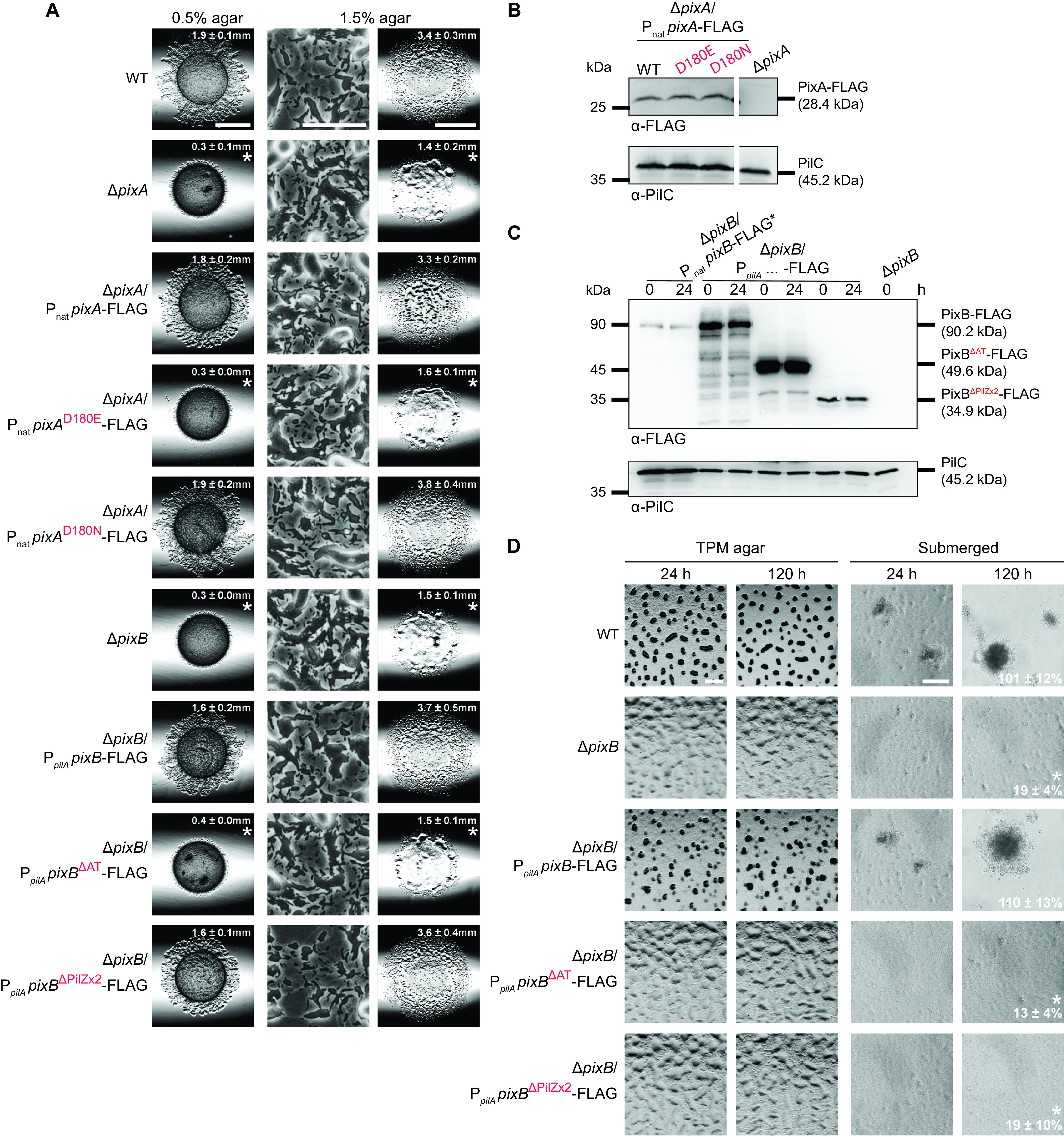
Role of the PixA receiver domain and PixB AT domain in regulation of motility and development. (A) Motility assays of strains expressing mutant variants of PixA and PixB. Motility was analyzed as for Fig. 2A. Bars, 3 mm (0.5% agar), 100 μm (1.5% agar, left), and 3 mm (1.5% agar, right). (B and C) Immunoblot detection of PixA-FLAG (B) and PixB-FLAG (C) variants. Samples were prepared and analyzed as for Fig. 6C and D. For panel B, samples were loaded on the same gel; the gap indicates lanes that were removed for presentation purposes. Ten micrograms of protein was loaded per lane except for lanes marked with asterisks, in which 100 μg of protein was loaded, and then samples were separated by SDS-PAGE. (D) Development assays in strains expressing mutant variants of PixB. Development was analyzed as for Fig. 2B. Bars, 500 μm (TPM agar) and 100 μm (submerged).

The receiver domain of PixA contains the conserved residues important for phosphorylation, including the putative phosphorylated residue Asp180 (Fig. S5). Mutation of the phosphorylatable Asp to Glu mimics the phosphorylated form in some response regulators (75, 76), while the Asp-to-Asn substitution blocks phosphorylation. Therefore, to test whether phosphorylation of the receiver domain could be important for PixA function, we ectopically expressed FLAG-tagged full-length PixA variants with a D180E or D180N substitution. Only PixA^WT^-FLAG and PixA^D180N^-FLAG restored motility, including the reversal frequency in the Δ*pixA* mutant, while PixA^D180E^-FLAG did not (Fig. 7A; Fig. S7C and D). PixA^WT^-FLAG, PixA^D180E^-FLAG, and PixA^D180N^-FLAG accumulated at similar levels during growth (Fig. 7B). Altogether, these findings support a scenario whereby PixA might be phosphorylated and in which the nonphosphorylated form of PixA would represent the active form of the protein.

To test which domains in PixB are important for function, we ectopically expressed PixB^WT^-FLAG as well as truncated variants that lacked either the AT domain (PixB^ΔAT^-FLAG) or the two PilZ domains (PixB^ΔPilZx2^-FLAG) in the Δ*pixB* mutant. Because the two truncated variants could not be detected by Western blotting when expressed from the native *pixB* promoter, all variants were expressed from the stronger *pilA* promoter. The two variants as well as the WT protein accumulated at a higher level than PixB^WT^-FLAG expressed from the native promoter during growth and development (Fig. 7C). Importantly, overexpression of PixB^WT^-FLAG restored both motility and development, while overexpression of PixB^ΔAT^-FLAG restored neither motility nor development (Fig. 7A and D; Fig. S7C and D). Notably, PixB^ΔPilZx2^-FLAG was sufficient to restore motility, but not development (Fig. 7A and D; Fig. S7C and D). Altogether, these observations support the idea that the AT activity is key to PixB function and that c-di-GMP binding to the PilZ domains is important for activating AT activity during development.

## DISCUSSION

Here, we report the identification of two PilZ domain proteins, PixA and PixB, that are important for regulation of motility in M. xanthus and, specifically, important for maintaining a correct cellular reversal frequency during growth. Moreover, PixB is important for completion of the developmental program that results in the formation of spore-filled fruiting bodies.

Similarly to the previously identified PilZ domain protein PlpA (66), PixA and PixB are not important for motility *per se* but rather are important for regulation of the cellular reversal frequency. Mutants lacking PixA or PixB accumulate EPS similarly to the WT. These observations argue that PixA and PixB are not involved in the known responses to altered c-di-GMP levels during growth, i.e., altered EPS synthesis and reduced *pilA* transcription. Lack of PlpA (66), PixA, or PixB causes an increase in the reversal frequency, suggesting that all three proteins function to inhibit reversals. Importantly, we observed in epistasis experiments that the three double mutants and the triple mutant had additive phenotypes compared to the three single mutants with respect to the reversal frequency. Lack of PlpA (66) and PixA causes hyperreversals independently of the Frz system, while the hyperreversals in the absence of PixB are Frz dependent. Altogether, these observations suggest a model in which PlpA, PixA, and PixB act in independent pathways to regulate and establish the correct cellular reversal frequency. Moreover, they support a model whereby PixB acts upstream of the Frz system and PixA and PlpA act in independent pathways downstream of the Frz system to inhibit reversals.

PixB contains two PilZ domain proteins as well as a C-terminal AT domain. PixB binds c-di-GMP *in vitro*; however, it is not clear which PilZ domain(s) is involved in this binding. By analyzing truncated variants of PixB or variants with single amino acid substitutions in the sequence motifs for c-di-GMP binding, we found that the AT domain was necessary and sufficient for correct reversals during growth. In contrast, the two PilZ domains alone did not complement the reversal defect in the Δ*pixB* mutant. Along the same lines, substitutions in the c-di-GMP binding motifs did not interfere with PixB activity in regulation of the reversal frequency under the conditions tested. These observations support a model whereby the AT activity is key to the effect of PixB on Frz-dependent reversals during growth, while the two PilZ domains and c-di-GMP binding are not important. How then would the AT domain act to inhibit reversals? Acetyltransferases of the GNAT family can acetylate amino groups of different substrates, including small molecules, peptidoglycan, nucleotide-linked monosaccharides, and proteins; however, the substrate cannot be determined based on protein sequence (77, 78). Therefore, further studies are required to determine the substrate of PixB.

While the AT domain of PixB is sufficient for correct reversals during growth, all three domains of PixB are important for completion of development. Similarly, PixB variants with single amino acid substitutions in the sequence motifs for c-di-GMP binding did not support development, strongly suggesting that c-di-GMP binding by PixB plays role in fruiting body formation. We previously showed that the level of c-di-GMP increases ∼10-fold during development (54). We speculate that the increased c-di-GMP level allows binding of c-di-GMP to the two PilZ domains to boost AT activity of PixB during development. Such a mechanism would be similar to that recently described for an AT from Mycobacterium tuberculosis that depends on cAMP binding to a cyclic nucleotide-binding domain to activate acetyltransferase activity (79). Interestingly, the cellular reversal frequency of WT cells decreases during development (80); thus, it is interesting to speculate that PixB activity is essential for this decrease. In future experiments, it will be of interest to determine the substrate of PixB and to elucidate how the two PilZ domains may regulate PixB activity. Of note, the primary defect in *dmxB* mutants, which lack the DGC responsible for the ∼10-fold increase in c-di-GMP during development, is reduced EPS synthesis (54). The Δ*pixB* mutant accumulates EPS during development at WT levels, arguing that the output of DmxB activity is not channeled only through PixB.

PlpA is a stand-alone PilZ domain protein that does not bind c-di-GMP *in vitro* (66). In contrast, PixA is a response regulator that binds c-di-GMP *in vitro*. Under the conditions tested, c-di-GMP binding by the PilZ domain was not important for regulation of motility. Moreover, genetic evidence supports the idea that the receiver domain could be phosphorylated and that the nonphosphorylated form represents the active form of the protein. The Frz-independent hyperreversals resulting from lack of PixA and PlpA are characteristic of mutants in which the localization of the small GTPase MglA, the key regulator of motility in M. xanthus, in its GTP-bound form is shifted from unipolar to more bipolar (47, 50, 74, 81, 82). Consistently, in Δ*plpA* (66) and Δ*pixA* mutants, MglA localization is shifted to more bipolar. These observations together with the epistasis experiments strongly support the idea that PixA and PlpA act in independent pathways at the level of the cell polarity system that establish unipolar T4P and Agl/Glt complex assembly at the leading cell pole and, in the case of the Agl/Glt complexes, their disassembly at the lagging pole. PlpA was reported to localize to the lagging cell pole in an MglB-dependent manner (66), while it is not known whether PixA is polarly localized. In future experiments, it will be interesting to test for interactions between PlpA, PixA, and proteins of the polarity module. Genetic evidence suggests that PixA activity could be regulated by phosphorylation of the receiver domain. Typically, partner proteins of two-component systems are encoded by neighboring genes (72); however, in M. xanthus, these proteins are often encoded by orphan genes (83). This is also the case for the *pixA* gene, where there is no neighboring gene encoding a histidine protein kinase. Therefore, it will also be of interest to identify the potential partner kinase of PixA.

Interestingly, Pseudomonas aeruginosa and Xanthomonas axonopodis pv. citri contain a stand-alone PilZ protein that stimulates T4P formation (61, 62). In contrast, in M. xanthus, PlpA, PixA, and PixB regulate T4P-dependent motility rather than T4P formation. We conclude that PilZ domain proteins can act at different levels to affect T4P-dependent motility.

## MATERIALS AND METHODS

### Cultivation of M. xanthus and E. coli.

All M. xanthus strains used in this study are derivatives of WT DK1622 (84). In-frame deletions were generated as described elsewhere (83). All M. xanthus strains generated were verified by PCR. M. xanthus strains, plasmids, and oligonucleotides used are listed in Table 1, Table 2, and Table S1 in the supplemental material, respectively. M. xanthus cells were grown at 32°C in 1% CTT medium (1% [wt/vol] Bacto Casitone, 10 mM Tris-HCl [pH 8.0], 1 mM K_2_HPO_4_-KH_2_PO_4_ [pH 7.6], 8 mM MgSO_4_) or on 1% CTT–1.5% agar plates at 32°C with addition of kanamycin (40 μg ml^−1^) or oxytetracycline (10 μg ml^−1^) (85). Escherichia coli cells were cultivated in LB (86) liquid medium with shaking or on LB 1.5% agar plates at 37°C with addition of kanamycin (40 μg ml^−1^) or tetracycline (10 μg ml^−1^). All plasmids were propagated in E. coli Top10 (Invitrogen Life Technologies) unless stated otherwise.

**TABLE 1:** M. xanthus strains used in this study

Strain	Characteristic(s)	Reference
DK1622	Wild type	84
DK10410	Δ*pilA*	97
SA5293	Δ*aglQ*	98
SA6462	Δ*mrpC*	This study
SA8802	Δ*frzE*	51
SA8185	*mglA-mVenus*	51
SA8047	Δ*pixA mglA-mVenus*	This study
SA8060	ΔMXAN_0063	This study
SA8034	ΔMXAN_0614	This study
SA8069	ΔMXAN_0833	This study
SA8087	ΔMXAN_0961	This study
SA8042	ΔMXAN_1087 (*pixA*)	This study
SA8082	ΔMXAN_1467 (*pkn1*)	This study
SA8509	ΔMXAN_2528 (*plpA*)	This study
SA8811	ΔMXAN_2604 (*pixB*)	This study
SA8800	ΔMXAN_2649	This study
SA8812	ΔMXAN_2902	This study
SA8506	ΔMXAN_3585	This study
SA8801	ΔMXAN_3721	This study
SA8088	ΔMXAN_3778	This study
SA8039	ΔMXAN_3788	This study
SA8508	ΔMXAN_4328	This study
SA8078	ΔMXAN_4567	This study
SA8049	ΔMXAN_5615	This study
SA8083	ΔMXAN_5655	This study
SA5634	ΔMXAN_5707	This study
SA8029	ΔMXAN_5804	This study
SA8035	ΔMXAN_6013	This study
SA5639	ΔMXAN_6605	This study
SA8050	ΔMXAN_6957	This study
SA8040	ΔMXAN_7024	This study
SA8054	Δ*pixA* Δ*frzE*	This study
SA10130	Δ*pixB* Δ*frzE*	This study
SA10131	Δ*pixA* Δ*plpA*	This study
SA10132	Δ*pixB* Δ*plpA*	This study
SA10134	Δ*pixA* Δ*pixB*	This study
SA10139	Δ*pixA* Δ*pixB* Δ*plpA*	This study
SA8045	Δ*pixA*/P_nat_ *pixA*	This study
SA10120	Δ*pixA*/P_nat_ *pixA*-FLAG MXAN_2604MXANMXANMXMXAN_1087	This study
SA10121	Δ*pixA*/P_nat_ *pixA*^R9A^-FLAG	This study
SA10136	Δ*pixA*/P_nat_ *pixA*^D180E^-FLAG	This study
SA10140	Δ*pixA*/P_nat_ pixA^D180N^-FLAG	This study
SA8829	Δ*pixB*/P_nat_ *pixB*	This study
SA9933	Δ*pixB*/P_nat_ *pixB-F*LAG	This study
SA9935	Δ*pixB*/P_nat_ *pixB*^R121A^-FLAG	This study
SA9936	Δ*pixB*/P_nat_ *pixB*^R331A^-FLAG	This study
SA9937	Δ*pixB*/P_nat_ *pixB*^R121A/R331A^-FLAG	This study
SA9941	Δ*pixB* /P*_pilA_ pixB*-FLAG	This study
SA9940	Δ*pixB* /P*_pilA_ pixB*^ΔAT^-FLAG	This study
SA10135	Δ*pixB* /P*_pilA_ pixB*^ΔPilXx2^-FLAG	This study

**TABLE 2:** Plasmids used in this study

Plasmid	Description	Reference or source
pBJ114	Kan^r^ *galK*	99
pSWU30	*attB* Tet^r^	100
pSW105	*attB* Kan^r^	101
pET24b(+)	Expression vector, Kan^r^	Novagen
pAP19	pBJ114, *frzE*, in-frame deletion, Kan^r^	49
pBJ114_*mrpC*	pBJ114, *mrpC*, in-frame deletion, Kan^r^	102
pLC20	pBJ114, *mglA-mVenus*, gene replacement at native site, Kan^r^	51
pSK55	pBJ114, MXAN_0063, in-frame deletion, Kan^r^	This study
pSK32	pBJ114, MXAN_0614, in-frame deletion, Kan^r^	This study
pSK56	pBJ114, MXAN_0833, in-frame deletion, Kan^r^	This study
pSK57	pBJ114, MXAN_0961, in-frame deletion, Kan^r^	This study
pSK41	pBJ114, *pixA*, in-frame deletion, Kan^r^	This study
pSK93	pBJ114, *pkn1*, in-frame deletion, Kan^r^	This study
pMP114	pBJ114, *plpA*, in-frame deletion, Kan^r^	This study
pES07	pBJ114, *pixB*, in-frame deletion, Kan^r^	This study
pES02	pBJ114, MXAN_2649, in-frame deletion, Kan^r^	This study
pES08	pBJ114, MXAN_2902, in-frame deletion, Kan^r^	This study
pMP115	pBJ114, MXAN_3585, in-frame deletion, Kan^r^	This study
pES01	pBJ114, MXAN_3721, in-frame deletion, Kan^r^	This study
pSK42	pBJ114, MXAN_3778, in-frame deletion, Kan^r^	This study
pSK94	pBJ114, MXAN_3788, in-frame deletion, Kan^r^	This study
pMP116	pBJ114, MXAN_4328, in-frame deletion, Kan^r^	This study
pSK58	pBJ114, MXAN_4567, in-frame deletion, Kan^r^	This study
pSK95	pBJ114, MXAN_5615, in-frame deletion, Kan^r^	This study
pSK96	pBJ114, MXAN_5655, in-frame deletion, Kan^r^	This study
pDJS82	pBJ114, MXAN_5707, in-frame deletion, Kan^r^	This study
pSK34	pBJ114, MXAN_5804, in-frame deletion, Kan^r^	This study
pSK36	pBJ114, MXAN_6013, in-frame deletion, Kan^r^	This study
pDJS94	pBJ114, MXAN_6605, in-frame deletion, Kan^r^	This study
pSK62	pBJ114, MXAN_6957, in-frame deletion, Kan^r^	This study
pSK37	pBJ114, MXAN_7024, in-frame deletion, Kan^r^	This study
pSK53	pSWU30, P_nat_ *pixA* *attB* Tet^r^	This study
pSK139	pSWU30, P_nat_ *pixA*-FLAG *attB* Tet^r^	This study
pSK140	pSWU30, P_nat_ *pixA*^R9A^-FLAG *attB* Tet^r^	This study
pSK144	pSWU30, P_nat_ *pixA*^D180E^-FLAG *attB* Tet^r^	This study
pSK145	pSWU30, P_nat_ *pixA*^D180N^-FLAG *attB* Tet^r^	This study
pES12	pSWU30, P_nat_ *pixB* *attB* Tet^r^	This study
pPK11	pSWU30, P_nat_ *pixB*-FLAG *attB* Tet^r^	This study
pPK13	pSWU30, P_nat_ *pixB*^R121A^-FLAG *attB* Tet^r^	This study
pPK14	pSWU30, P_nat_ *pixB*^R331A^-FLAG *attB* Tet^r^	This study
pPK15	pSWU30, P_nat_ *pixB*^R121A R331A^-FLAG *attB* Tet^r^	This study
pPK18	pSW105, P*_pilA_ pixB*-FLAG *attB* Kan^r^	This study
pPK17	pSW105, P*_pilA_ pixB*^ΔAT^-FLAG *attB* Kan^r^	This study
pSK143	pSW105, P*_pilA_ pixB* ^ΔPilZx2^ -FLAG *attB* Kan^r^	This study
pSK51	pET24b(+), *pixA*-His_6_ Kan^r^	This study
pSK141	pET24b(+), *pixA*^R9A^-His_6_ Kan^r^	This study
pES09	pET24b(+), *pixB*-His_6_ Kan^r^	This study
pPK21	pET24b(+), *pixB*^R121A^-His_6_ Kan^r^	This study
pES13	pET24b(+), *pixB*^R331A^-His_6_ Kan^r^	This study
pPK22	pET24b(+), *pixB*^R121A/R331A^-His_6_ Kan^r^	This study

### Motility assays.

Motility assay were done as described elsewhere, with modifications (37). Briefly, exponentially growing M. xanthus cells were harvested at 5,000 × *g* for 5 min and resuspended in 1% CTT to 7 × 10^9^ cells ml^−1^. From this suspension, 5 μl was spotted on 0.5% agar with addition of 1% CTT to a final concentration of 0.5% for T4P-dependent motility and on 1.5% agar with the addition of 1% CTT to a final concentration of 0.5% for gliding motility. Cells were incubated in the dark at 32°C for 24 h. Colony morphology was imaged using a Leica M205FA stereomicroscope with a Hamamatsu ORCA-flash V2 digital CMOS camera and a Leica DMi8 inverted microscope with a Leica DFC280 camera.

### Development.

M. xanthus development assays were performed as described previously (87) on solid TPM (10 mM Tris-HCl [pH 7.6], 1 mM K_2_HPO_4_-KH_2_PO_4_ [pH 7.6], 8 mM MgSO_4_) 1.5% agar plates and under MC7 buffer (10 mM MOPS [morpholinepropanesulfonic acid; pH 6.8], 1 mM CaCl_2_). Briefly, exponentially growing cells were harvested at 5,000 × *g* for 5 min and resuspended in MC7 buffer to 7 × 10^9^ cells ml^−1^. Then, 20 μl of cell suspension was spotted on TPM agar, and 50 μl was added to 350 μl of MC7 buffer in 24-well polystyrene plate (Falcon). At 24 h and 120 h, fruiting bodies were imaged with a Leica M205FA stereomicroscope with a Hamamatsu ORCA-flash V2 digital CMOS camera and a Leica DMi8 inverted microscope with Leica DFC280 camera. To determine sporulation efficiency, cells at 120 h of development were harvested from one 24-well polystyrene plate (Falcon). Cells were sonicated for 1 min (30% pulse; 50% amplitude with a UP200St sonifier and microtip; Hielscher) to disperse fruiting bodies and then incubated at 55°C for 2 h. Sporulation efficiency was calculated as the number of sonication- and heat-resistant spores formed after 120 h of development, relative to the WT. Spores were counted in a counting chamber (depth, 0.02 mm; Hawksley).

### Single-cell motility assays.

Assays were performed as described elsewhere (51). Briefly, to track individual cells moving by T4P-dependent motility, 5 μl of an exponentially growing cell culture was spotted on a 24-well polystyrene plate (Falcon) and incubated at room temperature (RT) for 10 min in the dark. Then, cells were covered with 500 μl of 1% methylcellulose in MMC buffer (10 mM MOPS [pH 7.6], 4 mM MgSO_4_, 2 mM CaCl_2_) and incubated in the dark at room temperature for 30 min. Cell movement was recorded for 10 min at 20-s intervals. For analysis, Metamorph (Molecular Devices) and ImageJ 1.52b (88) were used. For each cell, the distance moved per 20-s interval was determined and the speed per minute calculated; for reversals, the number of reversals per cell per 10 min was determined. Only cells that displayed movement were included in these analyses. To track individual cells moving by gliding, 5 μl of exponentially growing cultures was placed on 1.5% agar plates supplemented with 0.5% CTT, covered with a coverslip, and incubated at 32°C. After 4 h, cells were observed for 10 min at 20-s intervals at room temperature, and the speed per minute as well as the number of reversals per 10 min was calculated.

### Trypan blue dye binding assay.

The trypan blue dye binding assay was performed as described elsewhere (53). Briefly, overnight cultures were grown to a density of 7 × 10^8^ cells ml^−1^, centrifuged at 5,000 × *g* for 5 min, and resuspended in MC7 buffer to 7 × 10^9^ cells ml^−1^. A 20-μl portion of cell suspension was spotted on 0.5% agar supplemented with 0.5% CTT or on 1.5% agar plates supplemented with TPM buffer containing trypan blue at a final concentration of 10 μg/ml. Plates were incubated at 32°C in the dark for 24 h. Colonies were visualized using a plate scanner.

### Microscopy and analysis of fluorescence microscopy images.

Epifluorescence microscopy was performed as described elsewhere (51). Briefly, exponentially growing cells were placed on a thin 1.5% agar pad buffered with TPM buffer on a glass slide and immediately covered with a coverslip. After 30 min at 32°C, cells were visualized using a Leica DMi8 inverted microscope, and phase-contrast and fluorescence images were acquired using a Leica DFC280 camera. Cells in phase-contrast images were automatically detected using Oufti48. Fluorescence signals in segmented cells were identified and analyzed using a custom-made Matlab v2016b (MathWorks) script as described in reference 51. The script divides a cell into three regions: polar region 1, polar region 2, and the cytoplasmic region. The polar regions are defined as the parts of a cell within a distance of 10 pixels, corresponding to 0.64 μm, from a tip of a cell. The cytoplasmic region includes all pixels of the cell with the exception of the polar regions. A polar cluster was identified when three or more connected pixels within a polar region had a fluorescence signal higher than a cell-specific threshold signal of two standard deviations above the average fluorescence signal in the cytoplasmic region. The fluorescence of a polar cluster was defined as the sum of the fluorescence signal of all connected pixels that exceeded the threshold value in that polar region. The cytoplasmic signal was defined as the sum of the fluorescence signal of all pixels excluding the polar clusters. For each cell with a polar cluster(s), an asymmetry index (ω) was calculated as (total fluorescence at pole 1 − total fluorescence at pole 2)/(total fluorescence at pole 1 + total fluorescence at pole 2).

By definition, pole 1 is the pole with the higher fluorescence. ω varies between 0 (bipolar symmetric localization) and 1 (unipolar localization). The localization patterns were binned from the ω values as follows: unipolar (ω > 0.9), bipolar asymmetric (0.9 > ω > 0.2), and bipolar symmetric (ω < 0.2). Diffuse localization was determined when no polar signal was detected. For time-lapse epifluorescence microscopy, cells were prepared as described and recorded for 15 min, with images captured every 30 s. Data were processed with Metamorph 7.5 and ImageJ 1.52b.

### Immunoblot analysis.

Protein concentration was measured using a Bradford assay (Bio-Rad). Rabbit polyclonal anti-PilC (1:5,000 dilution) (89) and anti-FLAG (Rockland; 1:1,500 dilution) antibodies were used together with horseradish peroxidase (HRP)-conjugated goat anti-rabbit immunoglobulin G (Sigma-Aldrich) as a secondary antibody. Immunoblots were performed as described elsewhere (86). Blots were developed using Luminata Crescendo Western HRP substrate (Millipore) and visualized with a LAS-4000 luminescent image analyzer (Fujifilm).

### Protein purification.

To purify PixA-His_6_, PixA^R9A^-His_6_, PixB-His_6_, PixB^R121A^-His_6_ and PixB^R331A^-His_6_, PixB^R121A/R331^-His_6_, and PixB-His_6_, E. coli Rosetta 2 (DE3)/pLysS (Novagen) was transformed with pSK51, pSK141, pES09, pPK21, pES13, and pPK22, respectively. Cultures were grown in 1 liter of LB with addition of chloramphenicol and kanamycin at 37°C to an OD_600_ of 0.5 to 0.7. Protein expression was induced by addition of isopropyl-β-d-1-thiogalactopyranoside (IPTG) to a final concentration of 0.5 mM for 3 h at 37°C. Cells were harvested by centrifugation at 3,800 × *g* for 10 min at 4°C and resuspended in lysis buffer (50 mM NaH_2_PO_4_, 300 mM NaCl, 50 mM imidazole, 5% glycerol, and a cOmplete protease inhibitor cocktail tablet [Roche], pH 8.0). Next, cells were disrupted with a French press and centrifuged at 48,000 × *g* and 4°C for 40 min. Cleared cell lysate was filtered with a 0.45-μm sterile filter (Millipore Merck, Schwalbach, Germany) and loaded onto a 5-ml HiTrap chelating HP column (GE Healthcare) preloaded with NiSO_4_ as described by the manufacturer and pre-equilibrated in wash buffer (50 mM NaH_2_PO_4_, 300 mM NaCl, 50 mM imidazole, 5% glycerol [pH 8.0]). The column was washed with 20 column volumes of column wash buffer. Proteins were eluted with elution buffer A (50 mM NaH_2_PO_4_, 300 mM NaCl, 500 mM imidazole, 5% glycerol [pH 8.0]) using a linear imidazole gradient from 50 to 500 mM. Fractions containing purified His_6_-tagged proteins were combined and loaded onto a HiLoad 16/600 Superdex 75 pg (GE Healthcare) gel filtration column that was equilibrated with buffer (50 mM NaH_2_PO_4_, 300 mM NaCl, 5% glycerol [pH 6.5]). Fractions containing His_6_-tagged proteins were pooled, frozen in liquid nitrogen, and stored at −80°C.

### *In vitro* c-di-GMP binding assay.

c-di-GMP binding was determined using a DRaCALA assay with ^32^P-labeled c-di-GMP as described elsewhere (90, 91). ^32^P-labeled c-di-GMP was prepared by incubating 10 μM His_6_-DgcA with 1 mM GTP/[α-^32^P]GTP (0.1 μCi μl^−1^) in reaction buffer (total volume of 200 μl) overnight at 30°C. The reaction mixture was then incubated with 5 U of Antarctic phosphatase (New England Biolabs [NEB]) for 1 h at 22°C to hydrolyze unreacted GTP. The reaction was stopped by incubation for 10 min at 95°C. The reaction was centrifuged (10 min, 20,000 × *g*, 20°C), and the supernatant was used for the binding assay. ^32^P-labeled c-di-GMP was mixed with 20 μM protein and incubated for 10 min at RT in binding buffer (10 mM Tris [pH 8.0], 100 mM NaCl, 5 mM MgCl_2_). Fifteen microliters of this reaction mixture was transferred to a nitrocellulose filter, allowed to dry, and imaged using a STORM 840 scanner (Amersham Biosciences). For competition experiments, 0.4 mM unlabeled c-di-GMP (Biolog) or GTP (Sigma-Aldrich) was added.

### Bioinformatic analysis.

The KEGG SSDB (Sequence Similarity Database) (92) was used to identify homologs of the PilZ domain proteins in other fully sequenced *Myxococcales* genomes using a reciprocal best BLASTP hit method. Protein domains were identified using Pfam v33.1 (pfam.xfam.org) (93) and MyHits Motif scan (https://myhits.isb-sib.ch/cgi-bin/motif_scan) (94). Multiple alignment using Fast Fourier Transform from EMBL-EBI (95) was used to align protein sequences. Sequence identity and similarity were calculated using EMBOSS Needle software (96) (pairwise sequence alignment).
