# Supplementary material for: Three PilZ Domain Proteins, PlpA, PixA, and PixB, Have Distinct Functions in Regulation of Motility and Development in Myxococcus xanthus
Source: J Bacteriol. 2021 Jun 8;203(13):e00126-21. doi: 10.1128/JB.00126-21 (PMC8316039; doi:10.1128/JB.00126-21)
Supplement: Supplemental file 1 — Fig. S1 to S7, supplemental text, and Table S1. Download JB.00126-21-s0001.pdf, PDF file, 7.55 MB [file jb.00126-21-s0001.pdf]

Supplementary Information

Three PilZ domain proteins, PlpA, PixA and PixB, have distinct functions in regulation of motility and development in *Myxococcus xanthus*

Sofya Kuzmich[a *], Dorota Skotnicka[a *], Dobromir Szadkowski[a], Philipp Klos[a], María Pérez-Burgos[a], Eugenia Schander[a], Dominik Schumacher[a] & Lotte Søgaard-Andersen[a, #]

This file contains:

- Supplementary Figures 1-7
- Supplementary Materials and Methods
- Supplementary Table 1
- Supplementary References

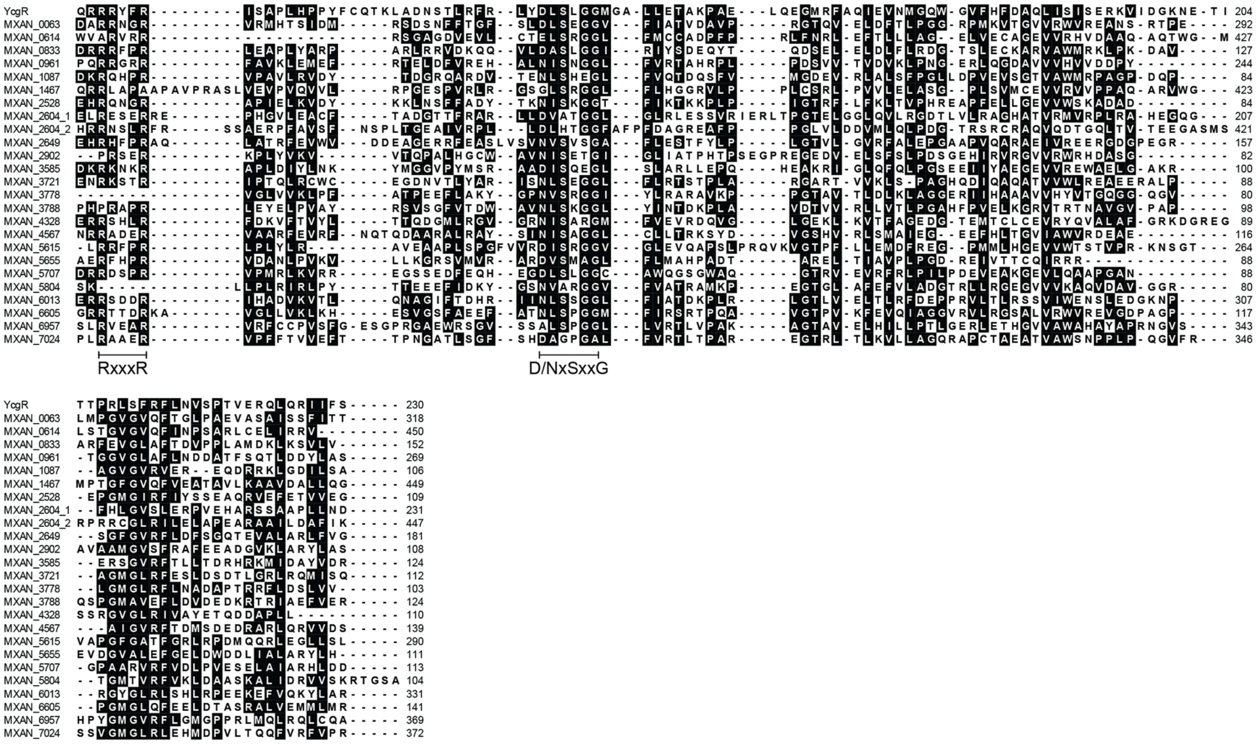

Figure S1. Sequence alignment of PilZ domains of *M. xanthus*.
Amino acid sequence of the *M. xanthus* PilZ domain were aligned with YcgR from *E. coli* (P76010) (1), which was also used as a reference to identify the residues important for c-di-GMP binding. The consensus sequence motifs for c-di-GMP binding are shown below. Identical and similar amino acids are marked on black.

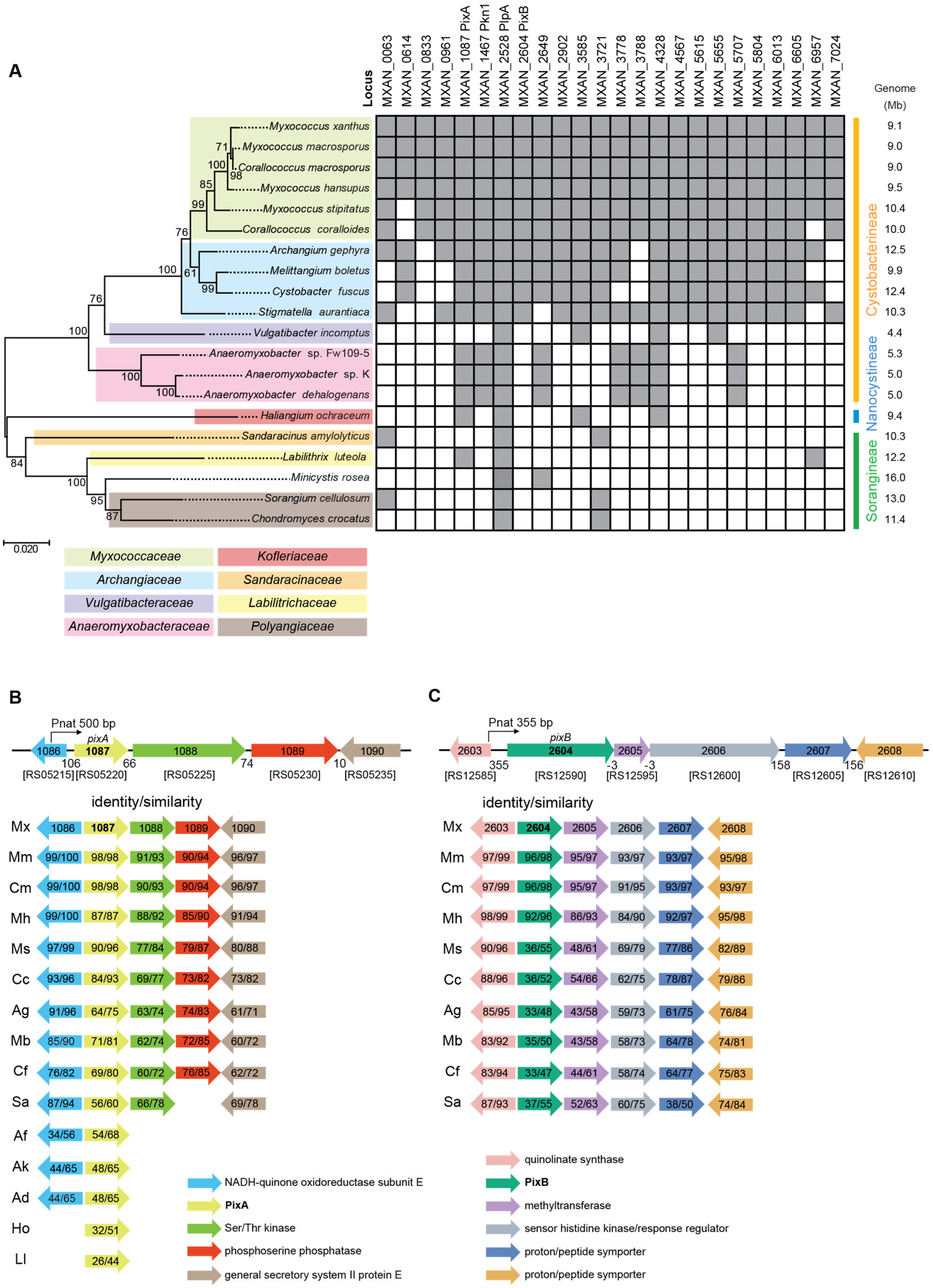
A
Locus
MXAN_0063
MXAN_0614
MXAN_0833
MXAN_0961
MXAN_1087 PixA
MXAN_1467 Pkn1
MXAN_2528 PlpA
MXAN_2604 PixB
MXAN_2649
MXAN_2902
MXAN_3585
MXAN_3721
MXAN_3778
MXAN_3788
MXAN_4328
MXAN_4567
MXAN_5615
MXAN_5655
MXAN_5707
MXAN_5804
MXAN_6013
MXAN_6605
MXAN_6957
MXAN_7024
Genome (Mb)
Myxococcus xanthus 9.1
Myxococcus macrosporus 9.0
Corallococcus macrosporus 9.0
Myxococcus hansupus 9.5
Myxococcus stipitatus 10.4
Corallococcus coralloides 10.0
Archangium gephyra 12.5
Melittangium boletus 9.9
Cystobacter fuscus 12.4
Stigmatella aurantiaca 10.3
Vulgatibacter incomptus 4.4
Anaeromyxobacter sp. Fw109-5 5.3
Anaeromyxobacter sp. K 5.0
Anaeromyxobacter dehalogenans 5.0
Haliangium ochraceum 9.4
Sandaracinus amylolyticus 10.3
Labilithrix luteola 12.2
Minicystis rosea 16.0
Sorangium cellulosum 13.0
Chondromyces crocatus 11.4
Cystobacterineae
Nanocystineae
Sorangineae
0.020
Myxococcaceae
Kofleriaceae
Archangiaceae
Sandaracinaceae
Vulgatibacteraceae
Labilitrichaceae
Anaeromyxobacteraceae
Polyangiaceae
B
Pnat 500 bp
pixA
1086 1087 1088 1089 1090
106 66 74 10
[RS05215] [RS05220] [RS05225] [RS05230] [RS05235]
identity/similarity
Mx 1086 1087 1088 1089 1090
Mm 99/100 98/98 91/93 90/94 96/97
Cm 99/100 98/98 90/93 90/94 96/97
Mh 99/100 87/87 88/92 85/90 91/94
Ms 97/99 90/96 77/84 79/87 80/88
Cc 93/96 84/93 69/77 73/82 73/82
Ag 91/96 64/75 63/74 74/83 61/71
Mb 85/90 71/81 62/74 72/85 60/72
Cf 76/82 69/80 60/72 76/85 62/72
Sa 87/94 56/60 66/78 69/78
Af 34/56 54/68
Ak 44/65 48/65
Ad 44/65 48/65
Ho 32/51
Ll 26/44
NADH-quinone oxidoreductase subunit E
PixA
Ser/Thr kinase
phosphoserine phosphatase
general secretory system II protein E
C
Pnat 355 bp
pixB
2603 2604 2605 2606 2607 2608
355 -3 -3 158 156
[RS12585] [RS12590] [RS12595] [RS12600] [RS12605] [RS12610]
identity/similarity
Mx 2603 2604 2605 2606 2607 2608
Mm 97/99 96/98 95/97 93/97 93/97 95/98
Cm 97/99 96/98 95/97 91/95 93/97 93/97
Mh 98/99 92/96 86/93 84/90 92/97 95/98
Ms 90/96 36/55 48/61 69/79 77/86 82/89
Cc 88/96 36/52 54/66 62/75 78/87 79/86
Ag 85/95 33/48 43/58 59/73 61/75 76/84
Mb 83/92 35/50 43/58 58/73 64/78 74/81
Cf 83/94 33/47 44/61 58/74 64/77 75/83
Sa 87/93 37/55 52/63 60/75 38/50 74/84
quinolinate synthase
PixB
methyltransferase
sensor histidine kinase/response regulator
proton/peptide symporter
proton/peptide symporter

Figure S2. Conservation of PilZ domain-containing proteins of *M. xanthus* in other myxobacteria.

A. Taxonomic distribution of *pilZ* genes in Myxococcales with fully sequenced genomes. A reciprocal best BlastP hit method was used to identify *pilZ* gene orthologs in myxobacteria. MXAN locus tags and gene names are indicated. Grey indicates conserved genes while white indicates that no homolog was identified. Left, 16S rRNA tree of Myxococcales with fully sequenced genomes was prepared as in (2). Bootstrap values (500 replicates) are shown next to the branches (3). Family and suborder classification are indicated. Genome size is indicated on the right.

B, C. Locus organization of *pixA* (B) and *pixB* (C). Top panel: arrows indicate direction of transcription. Distance between start and stop codon of neighboring genes is indicated. The kinked arrows indicate the fragments referred to as the native promoters (P_{nat}), which were used to express the genes ectopically from the *attB* site. Bottom panel: conservation of the loci in fully sequenced Myxococcales genomes. Numbers in the arrows indicate % identity/similarity between proteins from *M. xanthus* and their homologs. Species abbreviations can be taken from (A).

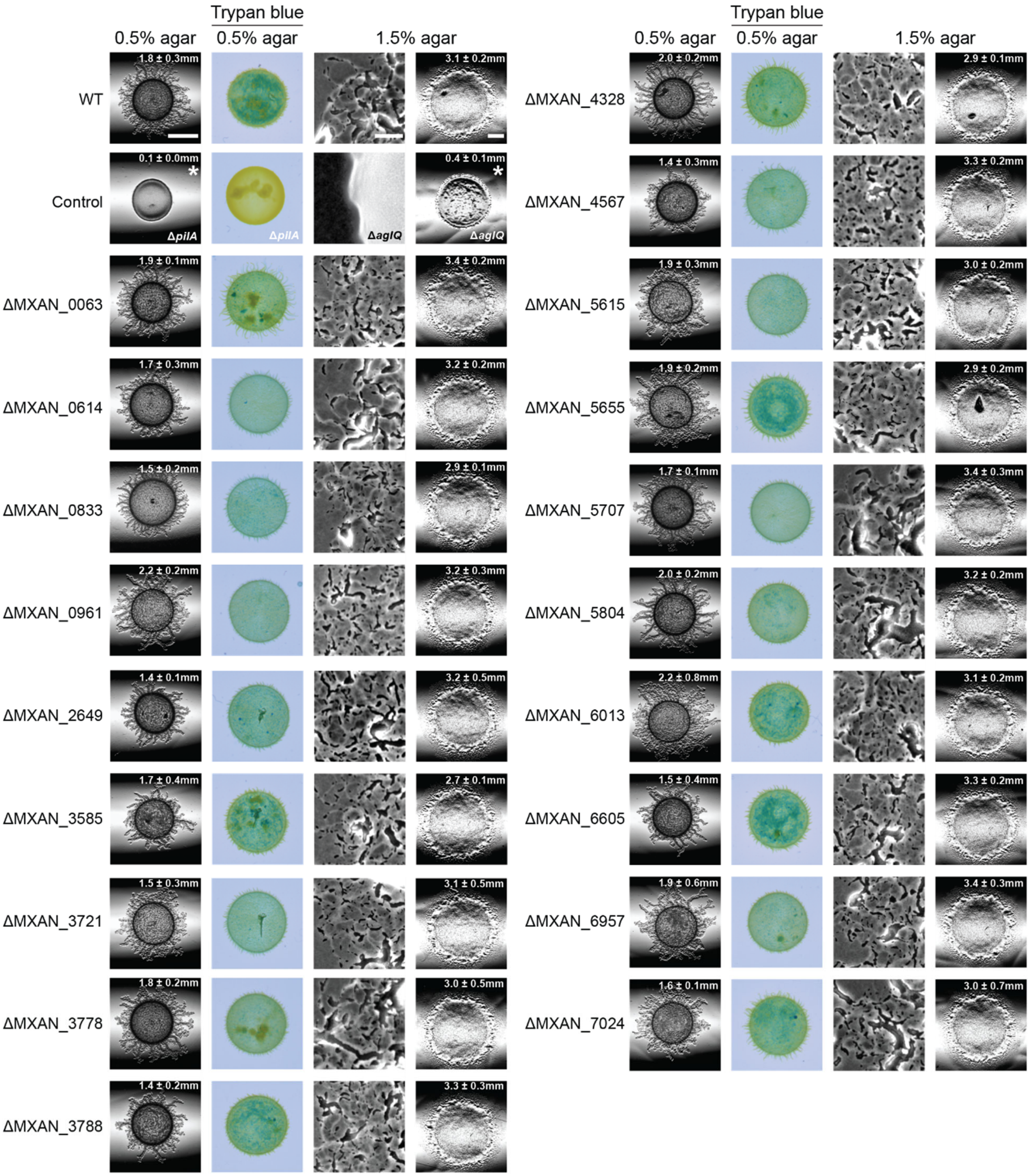

Figure S3. Motility assays for mutants lacking individual PilZ domain proteins. T4P-dependent motility and gliding motility were analyzed on 0.5% agar and 1.5% agar supplemented with 0.5% CTT, respectively. Motility was quantified by the increase in colony radius and numbers indicate the mean increase in colony radius ± standard deviation (SD) from three biological replicates after 24 h. *, $P < 0.05$ in a Student's t-test. Scale bars, 3 mm (0.5%

agar), 100 µm (1.5% agar, left), 3 mm (1.5% agar, right). EPS accumulation was determined on 0.5% agar supplemented with 0.5% CTT and 10 µg/ml Trypan blue.

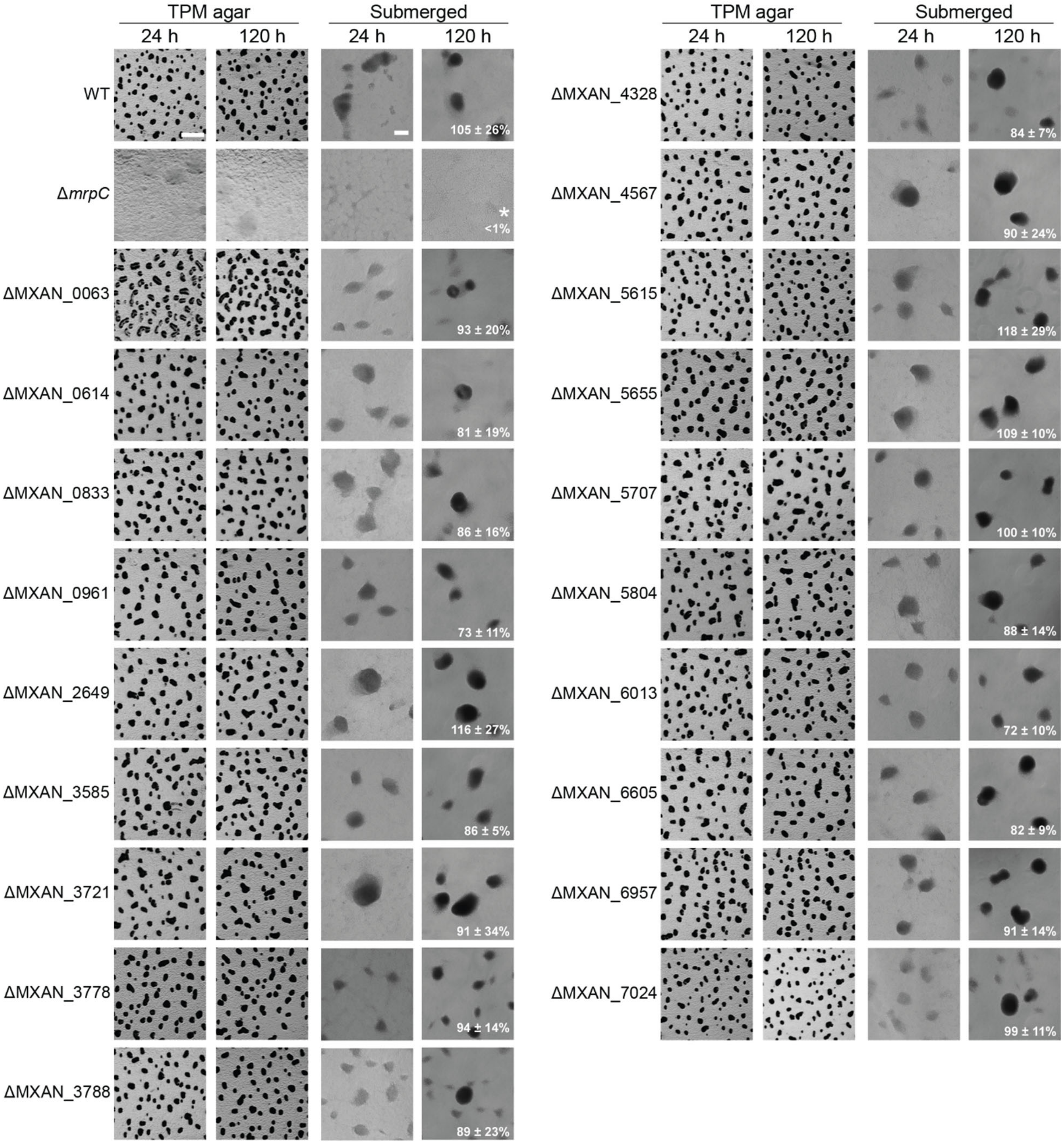

Figure S4. Development assays for mutants lacking individual PilZ domain proteins. Fruiting body formation and sporulation on TPM agar and in MC7 submerged culture. Numbers indicate heat- and sonication resistant spores formed at 120 h of starvation in submerged culture in percentage of WT (100%) ± SD from three biological replicates. *, $P < 0.05$ in Student's t test. Scale bars, 500 µm (TPM agar), 100 µm (submerged).

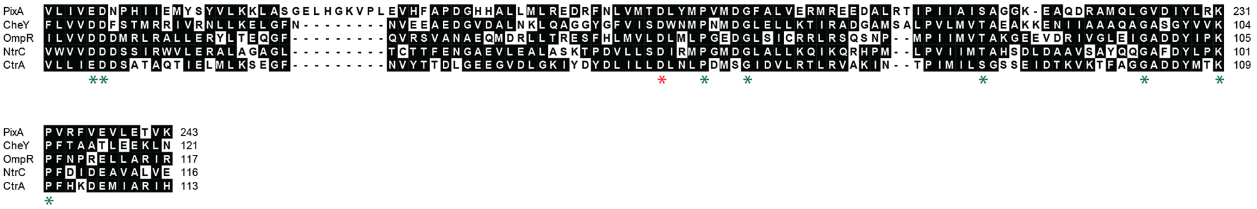

Figure S5. Sequence alignment of the receiver domain of PixA with receiver domains of NtrC (P0AFB8), OmpR (P0AA16), CtrA (B8H358) and CheY (P0AE67).
Identical and similar residues are marked on black. Residues important for phosphorylation (4) are marked with green asterisks and the phosphorylatable Asp residue is marked with a red asterisk.

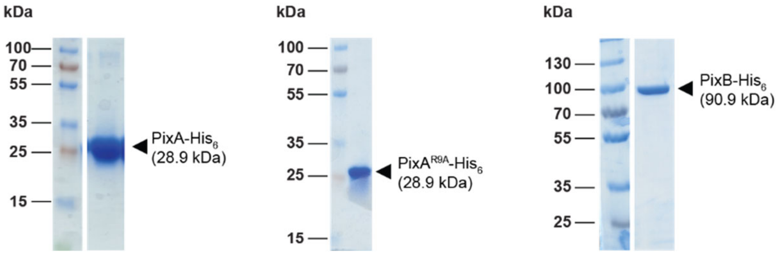

Figure S6. Purification of His6-tagged PixA and PixB variants.
Purified PixA-His$_6$ and PixB-His$_6$ protein variants were separated by SDS-PAGE and stained with Coomassie blue. Calculated molecular mass of the different proteins is indicated. Molecular size markers are indicated on the left.

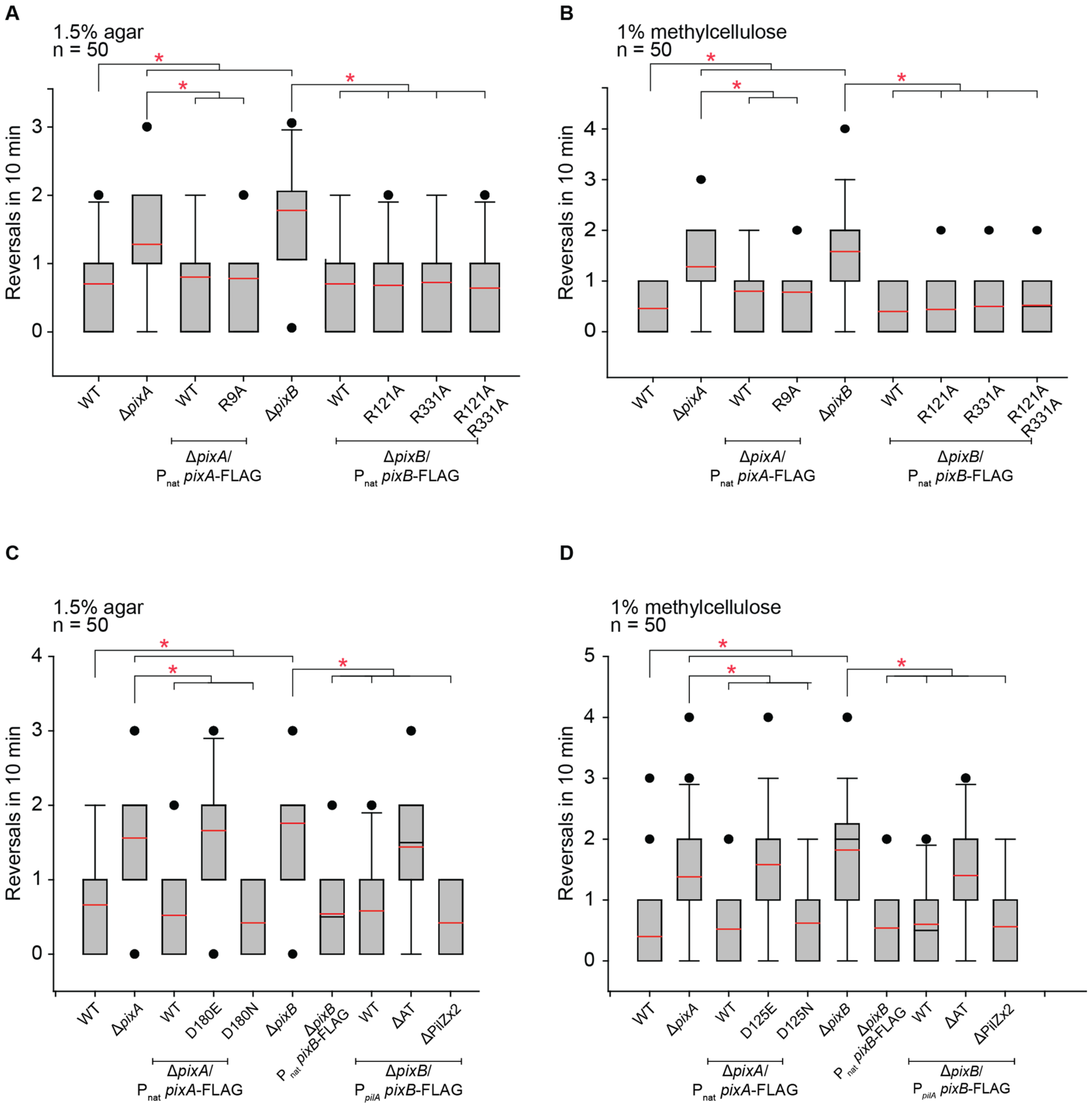

Figure S7. Single cell reversal frequency of strains of indicated genotypes.
A-D. Reversals of indicated strains on 1.5% agar supplemented with 0.5% CTT (A, C) or in 1% methylcellulose (B, D). Boxplots are as in Fig. 4D, F. * $P < 0.05$ in Mann-Whitney Rank Sum Test, n = 50 cells.

Supplementary Materials and Methods

Plasmid construction:

The plasmids pSK32, pSK34, pSK36, pSK37, pSK41, pSK42, pSK55, pSK56, pSK57, pSK58, pSK62, pSK93, pSK94, pSK95, pSK96, pMP114, pMP115, pMP116, pES01, pES02, pES07, pES08, pDJS82, pDJS94 were generated for the construction of the MXAN_0614, MXAN_5804, MXAN_6013, MXAN_7024, MXAN_1087 (*pixA*), MXAN_3778, MXAN_0063, MXAN_0833, MXAN_0961, MXAN_4567, MXAN_6957, MXAN_1467 (*pkn1*), MXAN_3788, MXAN_5615, MXAN_5655, MXAN_2528 (*plpA*), MXAN_3585, MXAN_4328, MXAN_3721, MXAN_2649, MXAN_2604 (*pixB*), MXAN_2902, MXAN_5707 and MXAN_6605 in-frame deletion mutants respectively. The upstream and downstream regions of the genes were amplified using primer pairs SK82-SK61 and SK62-SK63, SK036-SK037 and SK038-SK039, SK72-73 and SK74-75, SK76-SK77 and SK78-SK79, SK64-SK65 and SK66-SK67, SK68-SK69 and SK70-SK71, SK156- SK157 and SK158- SK159, SK144-SK145 and SK146-SK147, SK160-SK161 and SK162-SK163, SK164-SK165 and SK166-SK167, SK148-SK149 and SK150-SK151, SK253-SK254 and SK255-SK256, SK245-SK246 and SK247-SK248, SK152-SK261 and SK252-SK155, SK237-SK238 and SK239-SK240, 2528_A-2528_B and 2528_C-2528_D, 3585_A-3585_B and 3585_C-3585_D, 4328_A-4328_B and 4328_C-4328_D, 3721_A-3721_B and 3721_C-3721_D, 2649_A-2649_B and 2649_C-2649_D, 2604_A-2604_B and 2604_C-2604_D, 2902_A-2902_B and2902_C-2902_D, 5707_A-5707_B and 5707_C-5707_D, 6605_A-6605_B and 6605_C-6605_D. The AB and CD DNA fragments were fused by overlap PCR reaction. Resulting AD fragments were cloned into KpnI/XbaI or EcoRI/XbaI site of pBJ114.

Plasmid **pSK53** was used to complement Δ*pixA* mutant with WT version of *pixA* under the native promoter. To amplify *pixA* with the native promoter primers SK128-SK129 were used. The product was cloned into HindIII/XbaI sites of pSWU30.

Plasmid **pSK139** was used to complement Δ*pixA* mutant with full-length version of *pixA* fused to FLAG-tag under the native promoter. To amplify *pixA*-FLAG with the native promoter primers SK128 - SK384 were used. The product was cloned into HindIII/XbaI sites of pSWU30.

Plasmid **pSK140** was used to complement Δ*pixA* mutant with full-length version of *pixA*^{R9A} fused to FLAG-tag under the native promoter. To amplify *pixA*^{R9A}-FLAG with the native promoter primers SK128 - SK385 and SK386 - SK384 were used. Obtained fragments were fused during

second round of PCR amplification using primers SK128 - SK384. The product was cloned into HindIII/XbaI sites of pSWU30.

Plasmid **pSK144** was used to complement Δ*pixA* mutant with full-length version of *pixA*^{D180E} fused to FLAG-tag under the native promoter. To amplify *pixA*^{D180E}-FLAG with the native promoter primers SK64 – SK190 and SK189 - SK384 were used. Obtained fragments were fused during second round of PCR amplification using primers SK64 - SK384. The product was cloned into HindIII/EcoRI sites of pSWU30.

Plasmid **pSK145** was used to complement Δ*pixA* mutant with full-length version of *pixA*^{D180N} fused to FLAG-tag under the native promoter. To amplify *pixA*^{D180N}-FLAG with the native promoter primers SK64 – SK192 and SK191 - SK384 were used. Obtained fragments were fused during second round of PCR amplification using primers SK64 - SK384. The product was cloned into HindIII/EcoRI sites of pSWU30.

Plasmid **pES12** was used to complement Δ*pixB* mutant with WT version of *pixB* under the native promoter. To amplify *pixB* with the native promoter primers Pnat 2604 XbaI fw - 2604 KpnI rev were used. The product was cloned into XbaI/KpnI sites of pSWU30.

Plasmid **pPK11** was used to complement Δ*pixB* mutant with full-length version of *pixB* fused to FLAG-tag under the native promoter. To amplify *pixB*-FLAG with the native promoter primers Pnat 2604 XbaI fw - 2604 FLAG Rw were used. The product was cloned into HindIII/XbaI sites of pSWU30.

Plasmid **pPK13** was used to complement Δ*pixB* mutant with full-length version of *pixB*^{R121A} fused to FLAG-tag under the native promoter. To amplify *pixB*^{R121A}-FLAG with the native promoter primers Pnat 2604 XbaI fw - R121A PilZ1 and R121A PilZ1 2604 fw - 2604 FLAG Rw were used. Obtained fragments were fused during second round of PCR amplification using primers Pnat 2604 XbaI fw - 2604 FLAG Rw. The product was cloned into HindIII/XbaI sites of pSWU30.

Plasmid **pPK14** was used to complement Δ*pixB* mutant with full-length version of *pixB*^{R331A} fused to FLAG-tag under the native promoter. To amplify *pixB*^{R331A}-FLAG with the native promoter primers Pnat 2604 XbaI fw - R331A 2604 rev and R331A 2604 fw - 2604 FLAG Rw were used. Obtained fragments were fused during second round of PCR amplification using primers Pnat 2604 XbaI fw - 2604 FLAG Rw. The product was cloned into HindIII/XbaI sites of pSWU30.

Plasmid **pPK15** was used to complement Δ*pixB* mutant with full-length version of *pixB*$^{R121A/R331A}$ fused to FLAG-tag under the native promoter. To amplify MXAN_2604^{R331A}-FLAG with the native promoter primers Pnat 2604 XbaI fw - R331A 2604 rev and R331A 2604 fw - 2604 FLAG Rw were used. As a template **pPK13** was used. Obtained fragments were fused during second round of PCR amplification using primers Pnat 2604 XbaI fw - 2604 FLAG Rw. The product was cloned into HindIII/XbaI sites of pSWU30.

Plasmid **pPK18** was used to complement Δ*pixB* mutant with full-length version of *pixB* fused to FLAG-tag under the *pilA* promoter. To amplify *pixB*-FLAG primers 2604 XbaI fw – 2604 FLAG Rw were used. The product was cloned into HindIII/XbaI sites of pSW105.

Plasmid **pPK17** was used to complement Δ*pixB* mutant with the version of *pixB* with deleted AT domain and fused to FLAG-tag under the *pilA* promoter. To amplify *pixB*$^{\Delta AT}$-FLAG primers 2604 XbaI fw - 2604 ΔGNAT-FLAG (451) rev were used. The product was cloned into HindIII/XbaI sites of pSW105.

Plasmid **pSK143** was used to complement Δ*pixB* mutant with the version of *pixB* with deleted PilZ domains and fused to FLAG-tag under the *pilA* promoter. To amplify *pixB*$^{\Delta PilZx24}$-FLAG primers SK387 - 2604 FLAG Rw were used. The product was cloned into HindIII/XbaI sites of pSW105.

Plasmid **pSK51** was used to purify PixA-His$_6$ from *E.coli*. To amplify *pixA* primers SK122 and SK123 were used. The product was cloned into NdeI/XhoI sites of pET24b(+).

Plasmid **pSK141** was used to purify PixA^{R9A}-His$_6$ from *E.coli*. To amplify *pixA*^{R9A} primers SK122 and SK123 were used on **pSK140** as a template. The product was cloned into NdeI/XhoI sites of pET24b(+).

Plasmid **pES09** was used to purify PixB-His$_6$ from *E.coli*. To amplify *pixB* primers 2604 NdeI fw – 2604 HindIII –stop rev were used. The product was cloned into NdeI/HindIII sites of pET24b(+).

Plasmid **pPK21** was used to purify PixB^{R121A}-His6 from E.coli. To amplify PixB^{R121A} primers 2604 NdeI fw - R121A PilZ1 2604 rev and R121A PilZ1 2604 fw - 2604 HindIII –stop rev were used. Obtained fragments were fused during second round of PCR amplification using primers 2604 NdeI fw - 2604 HindIII –stop rev. The product was cloned into NdeI/HindIII sites of pET24b(+).

Plasmid **pES13** was used to purify PixB^{R331A}-His$_6$ from *E.coli*. To amplify *pixB*^{R331A} primers 2604 NdeI fw - R331A 2604 rev and R331A 2604 fw - 2604 HindIII –stop rev were used. Obtained fragments were fused during second round of PCR amplification using primers 2604 NdeI fw – 2604 HindIII –stop rev. The product was cloned into NdeI/HindIII sites of pET24b(+).

Plasmid **pPK22** was used to purify PixB$^{R121A/R331A}$-His6 from E.coli. To amplify PixB$^{R121A/R331A}$ primers 2604 NdeI fw - R121A PilZ1 2604 rev and R121A PilZ1 2604 fw - 2604 HindIII –stop rev were used on **pPK14** DNA. Obtained fragments were fused during second round of PCR amplification using primers 2604 NdeI fw - 2604 HindIII –stop rev. The product was cloned into NdeI/HindIII sites of pET24b(+).

Table S1. Primers used in this study

Name	Sequence (5‘-3‘)	Description
SK156_A	GCGCGGTACCGACCGCACCTTCGCGGACGT	In-frame deletion of MXAN_0063
SK157_B	TCAGTCCGGGAAGAACATTCCCCCGGACGGCCCCAC	
SK158_C	GTGGGGCCGTCCGGGGGAATGTTCTTCCCGGACTG	
SK159_D	GCGCAAGCTTGCTTCACGTCCTCCAGGCCG	
SK201_E	GGCATCCTCCTCAATACCCG	
SK202_F	GTGCAGTTCAGGTGGTGGAT	
SK203_G	AAGCAGGTTCGGAGATGCAG	
SK204_H	CGCTGAGGTCCAGTGAGAAA	
SK82_A	GCGCGAATTCCCGCGTGCGCGGAGACGC	In-frame deletion of MXAN_0614
SK61_B	CACCTCCGCCCGGGCGCGCATCACCCC	
SK62_C	CGCGCCCGGGCGGAGGTGGTGCTGTGA	
SK63_D	GCGCTCTAGACGAACCACCTGCCCGGCC	
SK085_E	ATCGAGCGGCGCACCTGC	
SK086_F	GGCACGTCTACGTGAAGATGGACG	
SK087_G	GGCGTGCCCATGTCGCAC	
SK088_H	CCGCCGCCGTTTCATCCT	
SK144_A	GCGCGAATTCAGGCCGGTGACGTTGAGG	In-frame deletion of MXAN_0833
SK145_B	CTATGAGTCGGCCGGGCCAGCCAGCCCGTGGCACA	
SK146_C	GTGTGCCACGGGCTGGCTGGCCCGGCCGACTCATA	
SK147_D	GCGCTCTAGACTCAGAGGGCGGGGCTCA	
SK176_E	ACCTCTCGTTGGGGGATGAC	
SK177_F	GCACGGGATGAAATGGTCG	
SK178_G	CTGGCTGGGTTCTGGGATTT	
SK179_H	GAGCACCGACTTGAGCTTGT	
SK160_A	GCGCGAATTCCCACGCGCGGGTCCGTCTCG	In-frame deletion of MXAN_0961
SK161_B	TCAGCCCACGCCACCCGCAAGACCGAAGACGGGCA	
SK162_C	GTGCCCGTCTTCGGTCTTGCGGGTGGCGTGGGCTG	
SK163_D	GCGCTCTAGATGGAGGGCGACCCGGGCATGGTGC	
SK180_E	TCCGGGGCGAGCGGGTTGGTGCC	
SK181_F	TCGGGGACGCCGGGCACACGCA	
SK182_G	TGGACCACCACCGCGTCGCC	
SK183_H	CCGCGGCCTCCGTGCCTCAA	
SK64_A	GCGCGAATTCTCTCTCGCAAGGTGAACT	In-frame deletion of MXAN_1087 (*pixA*)
SK65_B	GCGCAAGAGCTCTGGACCCGGGTTCAT	
SK66_C	GGTCCAGAGCTCTTGCGCATCAAGTAG	
SK67_D	GCGCTCTAGAGCCTCGTCCAGCAGCAGC	
SK102_E	CTGGGTCAGGCTTTCGTGGTGAT	
SK103_F	CATGGCCGAGCGCGAGCT	
SK104_G	GGTCCGGCTCGCACTTTCTT	
SK105_H	GGACCTCCAGGGGGACCTTT	
SK253_A	GCCGGGTACCAAGAAGGGCATCGAGTAGCGCACC	In-frame deletion of MXAN_1467 (*pkn1*)
SK254_B	TCAAGGTGACCGGGCCCTGCTGCTCACCTCGGGCAT	

SK255_C	ATGCCCGAGGTGAGCAGCAGGGCCCGGTCACCTTG	
SK256_D	GCCGTCTAGAGCCGGCGATGGAGGTGACCTT	
SK257_E	TGCCCGTGCGGTAATGGGAC	
SK258_F	GCAAGCGCGGCCTCGATAAG	
SK259_G	AACGCGCCCTTCGTCAAGGT	
SK260_H	CGCGATGGAGGCGTATCGGT	
2528_A	ATATGGTACCCTCCAAGCGGAACCAGCCGA	In-frame deletion of MXAN_2528 (*plpA*)
2528_B	CGGCTTGTTCTCTGGACCTGTCTTCTG	
2528_C	GGTCCAGAGAACAAGCCGCTGCACTCG	
2528_D	ATATTCTAGAACCCCACCCAAATGAGGCAG	
2528_E	AGCACGGTGACCCACAGCAG	
2528_F	CCTGGCAGGAAGCCATGAAA	
2528_G	AGAACGGGCGCGCGCCCATC	
2528_H	CTTCTCCGTCAGCTCGCCGC	
2604_A	ATCGGGTACCGCCAGGCTGTCTCCCACGAA	In-frame deletion of MXAN_2604 (*pixB*)
2604_B	GTCATCCATCAGCACTGCGCGCGTCGT	
2604_C	GCAGTGCTGATGGATGACAAGGACATC	
2604_D	ATCGTCTAGACGCAGGGCGGCGAGCAGCTT	
2604_E	ATGACGTCTGCCTTCGTGCT	
2604_F	CGGAACAGGTGGAACAGCTT	
2604_G	AGCTCACGGTGACGGAGGAG	
2604_H	TGGCGATGGCGCCGTAGATT	
2649_A	ATCGGGTACCTGTCCGGCAAGGGCTGGCGT	In-frame deletion of MXAN_2649
2649_B	GGAAACGGATGCCTTCTGGCCACCCTT	
2649_C	CAGAAGGCATCCGTTTCCGACCCTTGG	
2649_D	ATCGTCTAGACCATTTGCAGTCTTGTCATC	
2649_E	ATCGTCATCTGGAACGACTA	
2649_F	GATGTGGATGAAGAACTTCA	
2649_G	TTCACGCCGTTGATTGAGAT	
2649_H	TCCACCTCGTTGGGCAGGGA	
2902_A	ATCGGGTACCTGGGAGAAGGGCTCGCCGCC	In-frame deletion of MXAN_2902
2902_B	CAAGCCGTGGGCGTAGGCGGACACAGG	
2902_C	GCCTACGCCCACGGCTTGTGGAAAGGA	
2902_D	ATCGTCTAGACGGCCCTCGAACCGCAAGCT	
2902_E	AGCAGGTCCACCGTCGCGAG	
2902_F	CGCCGGGCATCATGTTGCAG	
2902_G	TGCTGCTCCAAGGCGAGACG	
2902_H	CCAGGAAGTAGAGCGCCAGC	
3585_A	ATCGGGTACCTCGTAGTGGACCTTGACGGT	In-frame deletion of MXAN_3585
3585_B	ACGGTCGACCCGCTTGTTCTTCCGCTT	
3585_C	AACAAGCGGGTCGACCGTCACGGCAAC	
3585_D	ATCGTCTAGAGCCGCTGTCATCCAAGAGGC	
3585_E	TGGTGCCGTCCACCAGCCGG	
3585_F	AGCGCCGCCGCGCCGTAGAG	
3585_G	CGACATCTACCTGAACAAGT	

3585_H	ATCATCTTGCGGTGACGATC	
3721_A	ATCGGGTACCTTCGTCAACGACGCGGAGGC	In-frame deletion of MXAN_3721
3721_B	CTTCACCTGGCTCAACCCGGAACCTTC	
3721_C	GGGTTGAGCCAGGTGAAGGTCGGCTGG	
3721_D	ATCGTCTAGACCATTGCCAATGGCGAAGGC	
3721_E	TCTTCCTGGGCAACATCCAC	
3721_F	TCCCCGCGCCTGTCCGCCTT	
3721_G	TTCCCACCCAGCTTCGGTGC	
3721_H	GCGAAGCCGTCCCAGGGTAT	
SK68_A	GCGCGAATTCGGAAGTTCTTCCGGGTGT	In-frame deletion of MXAN_3778
SK69_B	TCCGAACAAGACCGCCGCCTGATTCGA	
SK70_C	GCGGCGGTCTTGTTCGGAAGAGCCTGA	
SK71_D	GCGCTCTAGAAAGTTGGGTCCACCGCTG	
SK110_E	CACCTTCACCGCGTCCACGC	
SK111_F	GGGCTCCGCGCCGCAGAA	
SK112_G	TTCCCCCGGTTCCTCCCAAT	
SK113_H	ATGGAGGGCACCGTGTTGTG	
SK245_A	GCCGGGTACCCCGGACCTCATCAACGCCGGG	In-frame deletion of MXAN_3788
SK246_B	CTATTCGGGCGGGAGCTCGCCGCGGGGTTCGTACA	
SK247_C	TTGTACGAACCCCGCGGCGAGCTCCCGCCCGAATAG	
SK248_D	GCCGTCTAGAGCACGGGCGTGAGCGCGC	
SK249_E	ACGCCTGCCTCCCTGGTACA	
SK250_F	GTGACGCCGCAGGGACTCAT	
SK251_G	CGCTCCACGAACTCCGCGATA	
SK252_H	CAGTCCCAACACCTCGACCCC	
4328_A	ATCGGGTACCATCGCCCGGTGAGGTGACGC	In-frame deletion of MXAN_4328
4328_B	GCGGTCGACACGGCGCTCGTGAGGACG	
4328_C	GAGCGCCGTGTCGACCGCGAGCGGGTG	
4328_D	ATCGTCTAGAGCGCCCGCCTGCTTGCGGTG	
4328_E	GAGGCGCTCCAGTACATCGC	
4328_F	CCGCCAACATCCTGTCGCAG	
4328_G	ACCTCCGTTTCGACAAGGTC	
4328_H	GTCGTCCTGCGTCTCATAGG	
SK164_A	GCGCGGTACCGCTGGGTGACGTCATTCTCC	In-frame deletion of MXAN_4567
SK165_B	CTATCGCTTGAAGCTGTCGCTGCTTCCCACACCCAC	
SK166_C	GTGGGTGTGGGAAGCAGCGACAGCTTCAAGCGATA	
SK167_D	GCGCTCTAGACTGATGCGCCGGCCTTCCGT	
SK197_E	CGGCTTCATTCCCATTCCCA	
SK198_F	GGACACCGAAGTGACGAACT	
SK199_G	GTGCGTATGACAACCAAGGC	
SK200_H	GTGGCTTCACCGAAATCGAC	
SK152_A	GCGCGAATTCCCCATCCGCCGGCCGGAGCA	In-frame deletion of MXAN_5615
SK261_B1	TCACGGCATCCGCGCGACCAGGGGGGAACTCAAGT	
SK262_C1	AACTTGAGTTCCCCCCTGGTCGCGCGGATGCCGTGA	
SK155_D	GCGCTCTAGAGGGCGTGCTGGGGGGCATGA	

SK172_E	CAGCTCGATGACCTTCACCAT	
SK173_F	GAGTACGCCATCGAGTCTTCC	
SK174_G	GCAAAGTGTCAGTCGCGTG	
SK175_H	GAGTGTTCATCGGCGGAGTAG	
SK237_A	GCCGGGTACCCCACGCCTCCTGGTTGCCCGC	In-frame deletion of MXAN_5655
SK238_B	TCAGGGCAGGCGCGGGTGGTTTCTCCTCTTCTCCAC	
SK239_C	GTGGAGAAGAGGAGAAACCACCCGCGCCTGCCCTG	
SK240_D	GCCGTCTAGATTGCATCCTCCCTGGTGCGGC	
SK241_E	AGCACCGTGCCGAAGTCGTT	
SK242_F	TTGGGCAATGGCCCGTGTGA	
SK243_G	GACGCTCGGTGATGGTTCGC	
SK244_H	GGCGAATCTGGCAGGTGGTGA	
SK352_R	GCACCACTCCGCTTCATTCG	
5707_A	ATCGGGTACCCAGGCACGAGGTGCCCCGAGA	In-frame deletion of MXAN_5707
5707_B	GCTCTGCACGCTCATGCTCGCGCCACC	
5707_C	AGCATGAGCGTGCAGAGCGGCACGGCC	
5707_D	ATCGTCTAGAAGTCAGGAGCAAATCCACGGC	
5707_E	GAACTTCGGACGCGCACTCTA	
5707_F	AGAGCGTCTGACAAGCGTGGA	
5707_G	GACTCGATGAGCGACAAGGCC	
5707_H	GGCCTGGAGCACCTCGCCCTT	
SK036_A	GCGCGGTACCAGGCTCGCGATGTGAATG	In-frame deletion of MXAN_5804
SK037_B	GCCGAAGAGGGACGCTGTCCCGCTCAT	
SK038_C	ACAGCGTCCCTCTTCGGCCGCCGCTGA	
SK039_D	GCGCTCTAGACTGGCGGCGGAGATGCTG	
SK050_ E	GGGACGACGGGCTACACCTGTTGC	
SK051_F	TTCTCGTATCGCGCCTATGTGGAGT	
SK052_G	AGTCCGACGCCGAAGCAGGC	
SK053_H	CACGCTCACGCCCAGGCTCTCTT	
SK72_A	GCGCGAATTCGTTCCTGGACCTGTTCAT	In-frame deletion of MXAN_6013
SK73_B	CTTCTTCAGGGCCGGCAGGCTCTGCAT	
SK74_C	CTGCCGGCCCTGAAGAAGAAGCCCTGA	
SK75_D	GCGCTCTAGAGGGCAGCGGCCCACCGCG	
SK089_E	CGAGGCTGCGGACGGTGA	
SK090_F	TCAGGGCCAGCGTGCGCT	
SK091_G	AGCCTGGACGAGCTGCGTCT	
SK092_H	AATTCTCCCAGATGACGGAGCTGCG	
6605_A	ATCGGAATTCACTCGCCGCTCTTGCGCTGGT	In-frame deletion of MXAN_6605
6605_B	CTTCTTCACGTCCGTGGTGCGCCTGCC	
6605_C	ACCACGGACGTGAAGAAGGCGCTGGGC	
6605_D	ATCGTCTAGACAGTAGAAGCCGTCCAACCTG	
6605_E	TGTGAACCACGCCAGGCTGCC	
6605_F	GCAGTGCCCGTCCTCGCGCTT	
6605_G	AAGGGCTTCCCCAAGTGCGCC	
6605_H	TTGAGCTCGCGGTTGAGACCG	

SK148_A	GCGCGAATTCACCACAACCACTGCCCCACC	In-frame deletion of MXAN_6957
SK149_B	TCACGGGCGCACCTCCGCTCCTGGAGCCGTCATCAT	
SK150_C	ATGATGACGGCTCCAGGAGCGGAGGTGCGCCCGTG	
SK151_D	GCGCTCTAGAACCGATGAGGATGCGCAGCG	
SK168_E	ATTCCGACGCACAACCTCAA	
SK169_F	CCGGTGGACCTGAAGGATTT	
SK170_G	CCACGAGCGAATCCTGGTT	
SK171_H	CGCTGTAGAGGATGCGATGA	
SK76_A	GCGCGAATTCGGTCGTCTTCGAGCTGTC	In-frame deletion of MXAN_7024
SK77_B	TGAGGCGCGGTTGGGAGGAGGGCTCAT	
SK78_C	CCTCCCAACCGCGCCTCAGGTTTCTGA	
SK79_D	GCGCTCTAGATTGTTCGTGCCCGTGTAG	
SK106_E	GCGAGGTGGTGCTGAAGC	
SK107_F	CACGAACGGTGGATGTAGAGC	
SK108_G	CCGACGACTTCCTGCCCAAG	
SK109_H	GGAACCAGTCGCCCCAGAAC	
SK128	GCGCTCTAGAAGTACCGCACGAGGCCAG	Complementation of Δ*pixA*
SK129	GCGCAAGCTTCTACTTGATGCGCAAGAGCTG	
SK384	GCCGAAGCTTTCACTTGTCGTCGTCGTCCTTGTAGTC CTTGATGCGCAAGAGCTG	*pixA*-FLAG
SK385	CTTGTTATGAACCCGGGTCCAGAGGACAAG**GCC**CAG CACCCCCGCGTCCCGGCCGTGCTGAG	R9A point mutation in *pixA*
SK386	CTCAGCACGGCCGGGACGCGGGGGTGCTG**GGC**CTT GTCCTCTGGACCCGGGTTCATAACAAG	
SK189	GGTGATGACG**GAG**CTCTACATGC	D180E point mutation in *pixA*
SK190	GCATGTAGAG**CTC**CGTCATCACC	
SK191	GGTGATGACG**AAC**CTCTACATGC	D180N point mutation in *pixA*
SK192	GCATGTAGAG**GTT**CGTCATCACC	
Pnat 2604 XbaI fw	ATCGTCTAGAGGCACCTCCCGACTCCCACC	Complementation of Δ*pixB*
2604 KpnI rev	ATCGGGTACCTCATGCCGCGATGTCCTTGT	
2604 XbaI fw	ATCGTCTAGAATGACCGTGACGACGCGCGC	
2604 FLAG Rw	GCGAAGCTTTCACTTGTCGTCGTCGTCCTTGTAGTCT GCCGCGATGTCCTT	*pixB*-FLAG
R121A PilZ1 2604 fw	GAATTGCTGGAACTG**GCG**GAGTCCGAGCGCCGC	R121A point mutation in *pixB*
R121A PilZ1 2604 rev	GCGGCGCTCGGACTC**CGC**CAGTTCCAGCAATTC	
R331A 2604 fw	ACCACCGC**GCG**AACAGCCTG	R331A point mutation in *pixB*
R331A 2604 rev	CAGGCTGTT**CGC**GCGGTGGT	

2604 GNAT-FLAG (451) rev	gcgaagctttcacttgtcgtcgtcgtccttgtagtcACACCGTGCCTTGAT	Deletion of AT domain in *pixB*
pSK143	GCCGTCTAGACAGCGGCTGGGCAATGGCAT	Deletion of both PilZ domains in *pixB*
SK122	gcgcCATATGaacccgggtccagaggac	Purification of *pixA*-His_6
SK123	gcgcCTCGAGcttgatgcgcaagagctgc	
2604 NdeI fw	ATCGCATATGATGACCGTGACGACGCGCGC	Purification of *pixB*-His_6
2604 HindIII -stop rev	ATCGAAGCTTTGCCGCGATGTCCTTGTCAT	

* Restriction sites are underlined and mutations introduced by site-directed mutagenesis are in bold.

Supplementary References

1. Amikam D, Galperin MY. 2006. PilZ domain is part of the bacterial c-di-GMP binding protein. Bioinformatics 22:3-6.

2. Pérez-Burgos M, García-Romero I, Valvano MA, Søgaard-Andersen L. 2020b. Identification of the Wzx flippase, Wzy polymerase and sugar-modifying enzymes for spore coat polysaccharide biosynthesis in *Myxococcus xanthus*. Mol Microbiol 113:1189-1208.

3. Felsenstein J. 1985. Confidence-limits on phylogenies - an approach using the bootstrap. Evolution 39:783-791.

4. Stock AM, Robinson VL, Goudreau PN. 2000. Two-component signal transduction. Annu Rev Biochem 69:183-215.
